# Plant species identity and plant-induced changes in soil physicochemistry—but not plant phylogeny or functional traits - shape the assembly of the root-associated soil microbiome

**DOI:** 10.1093/femsec/fiad126

**Published:** 2023-10-10

**Authors:** Alexa-Kate Byers, Leo M Condron, Maureen O'Callaghan, Lauren Waller, Ian A Dickie, Steve A Wakelin

**Affiliations:** Bioprotection Aotearoa, Lincoln University, PO Box 85084, Lincoln 7647, New Zealand; Bioprotection Aotearoa, Lincoln University, PO Box 85084, Lincoln 7647, New Zealand; AgResearch Ltd, 1365 Springs Road, Lincoln 7674, New Zealand; Biosecurity New Zealand, Ministry for Primary Industries, 34-38 Bowen Street, PO Box 2526, Wellington 6140, New Zealand; Bioprotection Aotearoa, School of Biological Sciences, University of Canterbury, PO Box 4800, Christchurch 8140, New Zealand; Ecology and Environment, Scion Research Ltd, 10 Kyle Street, Riccarton, Christchurch 8011, Canterbury, New Zealand

**Keywords:** microbial assembly, microbial selection, plant effects, plant phylogeny, root-associated soil microbiome

## Abstract

The root-associated soil microbiome contributes immensely to support plant health and performance against abiotic and biotic stressors. Understanding the processes that shape microbial assembly in root-associated soils is of interest in microbial ecology and plant health research. In this study, 37 plant species were grown in the same soil mixture for 10 months, whereupon the root-associated soil microbiome was assessed using amplicon sequencing. From this, the contribution of direct and indirect plant effects on microbial assembly was assessed. Plant species and plant-induced changes in soil physicochemistry were the most significant factors that accounted for bacterial and fungal community variation. Considering that all plants were grown in the same starting soil mixture, our results suggest that plants, in part, shape the assembly of their root-associated soil microbiome via their effects on soil physicochemistry. With the increase in phylogenetic ranking from plant species to class, we observed declines in the degree of community variation attributed to phylogenetic origin. That is, plant-microbe associations were unique to each plant species, but the phylogenetic associations between plant species were not important. We observed a large degree of residual variation (> 65%) not accounted for by any plant-related factors, which may be attributed to random community assembly.

## Teaser

Laboratory gut bacterial communities are not representative for natural field conditions.

## Introduction

The functional activities of the root-associated soil microbiome are of fundamental importance for plant health. The root-associated soil microbiome influences plant nutrient acquisition, pathogen defense, induced systemic resistance, growth, drought tolerance, and other traits (Berendsen et al. 2012, Philippot et al. 2013, Pieterse et al. 2014). Given the importance of the microbiome to plant fitness and health, soil fertility, and ecosystem productivity, defining the key processes that shape microbial assembly has been an ongoing pursuit within microbial ecology (Marschner et al. 2001, Lundberg et al. 2012, Pérez-Jaramillo et al. 2016, Fitzpatrick et al. 2018). Whilst many studies have performed observational research to describe patterns of microbial assembly and community ecology (Prosser 2020), fewer studies have performed direct manipulations in controlled experiments to examine the factors shaping root-associated soil microbial assembly during a plant’s early phases of growth and establishment.

Several studies have proposed that plant species (PS) themselves are the primary determinants of their associated microbiome (Becklin et al. 2012, Mendes et al. 2013, Bouffaud et al. 2014, Chaparro et al. 2014). Theoretically, as the phylogenetic relatedness of PS influences their degree of shared developmental and functional traits, it may also influence the phylogenetic similarity of the microorganisms that they recruit. Thus, with increasing phylogenetic similarity among PS, one may observe an increased relatedness of their microbiome. Findings supporting this hypothesis have been observed in several studies (Bouffaud et al. 2014, Lambais et al. 2014, Lei et al. 2019, Hartman et al. 2023). In contrast, other studies have observed that the abiotic conditions of the soil environment (i.e. soil type, pH, nutrient availability, and C:N ratio) are of greater influence on microbial community assembly in the rhizosphere and root-associated soil environment (Girvan et al. 2003, Ulrich and Becker 2006, Lauber et al. 2009, Xiao et al. 2017, Yeoh et al. 2017, Veach et al. 2019, Ren et al. 2020).

This ecological conundrum draws parallels to the nature-versus-nurture debate that has shaped research around human development for decades. Several studies have proposed an assembly model more akin to nature-via-nurture, whereby both the PS and soil shape the microbiome, and the relative strength of these different drivers will vary depending on the specific ecological context (Garbeva et al. 2008, Berg and Smalla 2009, Tkacz et al. 2015, Müller et al. 2016, Lee and Hawkes 2020). In the nature-via-nurture model, soil provides the primary source of microbial inoculum available to plants and sets the boundaries from which plants may select their microbiome. The dominant influence of soil type and edaphic properties on determining the broad patterns of microbial biogeography was recognized by Fierer and Jackson (2006) and Lauber et al. (2009). However, throughout their development and life span, plants and their root systems exert species-specific influences on the rhizosphere and root-associated soil environment, which drives environmental filtering of their microbiome (Berg and Smalla 2009, Chaparro et al. 2014, Reinhold-Hurek et al. 2015, Hu et al. 2018). Additionally, the symbiotic associations of plant hosts (e.g. N_2_-fixing rhizobia, arbuscular mycorrhizal fungi) have been identified to shape the assembly of the root microbiome (Hartman et al. 2023).

There have been two primary processes proposed that shape microbiomes: deterministic and stochastic (Goss-Souza et al. 2017). Niche-based, deterministic models propose that the biotic and abiotic conditions of the local environment drive microbial selection (Carroll et al. 2011, Goss-Souza et al. 2017). Deterministic models can be further split into primary and secondary processes. Primary deterministic processes constitute a more direct mechanism, whereby the release of plant-specific rhizo-deposits selects or favours microbial taxa from the wider soil microbial community (Hu et al. 2018, Sasse et al. 2018, Zhalnina et al. 2018). Secondary deterministic processes function indirectly whereby plant roots modify the general rhizosphere and soil conditions (pH, available P, nitrogen, etc.), and these changes, in turn, encourage the growth of microorganisms best adapted to that modified habitat space (Hinsinger 2001, Liang et al. 2002, Bell et al. 2015, van Veelen et al. 2020, Hernández-Cáceres et al. 2022). In contrast to deterministic models, stochastic models propose an element of randomness to community assembly (Dini-Andreote et al. 2015, Goss-Souza et al. 2017). These deterministic and stochastic processes do not occur independently of each other, and the challenge is determining the relative contribution of these under different experimental and ecological contexts.

We performed a plant experiment whereby a broad phylogenetic range of 37 different PS were grown in an identical blended soil medium. Following 10 months of growth, the root-associated soil microbiome was characterized using the 16S rRNA gene and ITS region sequencing. The differences in the structural variance of the root-associated soil microbiome between PS were related to their phylogenetic and functional traits, as well as to any plant-induced changes in soil physicochemistry (SC) that occurred throughout the experiment. By performing this experiment, our research aimed to partition the influences of primary and secondary deterministic processes (i.e. direct and indirect plant effects) and stochastic processes on microbial assembly in the root-associated soil environment. Furthermore, we hypothesize that the phylogenetic relatedness of the PS will be positively correlated to the phylogenetic similarity of their root-associated soil microbiome.

## Materials and methods

### Plant and soil sample collection

Plants from 37 different species were grown in a blended soil media or obtained from a commercial nursery (Southern Woods Plant Nursery, New Zealand). The selected PS covered a broad range of phylogenetic groups and included representatives from three plant classes, 12 orders, 14 families, and 31 genera. The PS covered a range of different life spans (annual, perennial, or long-lived), functional groups (e.g. grass, shrub, or tree), and provenances (exotic or native to Aotearoa New Zealand). They also included species with different mycorrhizal associations (arbuscular, AMF, ectomycorrhizal, EMF, and no association) and N_2_ fixation (presence or absence). Plant metadata was primarily obtained from PS profiles on the New Zealand Plant Conservation Network (https://www.nzpcn.org.nz/) and from literature searches where additional information was needed. The full list of PS used in this study and their associated metadata are provided in Supplementary Table S1.

The seeds or cuttings of each PS were planted in individual 10-L pots containing a blend of field-collected live soils mixed with a pasteurized soil: sand carrier for bulk. Between 12 and 20 replicate pots were established for each PS. The collection of the live soils was conducted across 12 sub-alpine, grass, and shrub-dominated sites that included the ranges of the plants being experimentally evaluated. This sampling design was performed to allow for the microbiomes associated with these species to be available for plant ‘recruitment’. However, it is important to state that we did not examine the microbial background of these ‘live’ soils before setting up the experiment. Details regarding the collection, handling, treatment, and mixing of these soils were first provided by Wakelin et al. (2021). The plants were randomized within a glasshouse and grown with regular watering and supplemental lighting when required. No fertilizers or other chemicals were added to the pots, and weeds were removed when apparent.

After 10 months of plant growth, root-associated soil samples were collected from each plant pot. Samples were collected from between four and seven replicate pots of each plant depending on plant availability [i.e. plants that had grown to full health and were not required for other research (Wakelin et al. 2021)]. All root-associated soil samples were collected aseptically by pressing an open 50-mL conical centrifuge tube into the soil adjacent to the stem(s) of the plant in each pot directly into the root zone. Three samples from around the circumference of each pot were collected and pooled to provide a single sample for each replicate of each PS. Pooled soil samples were sieved to 2 mm and stored at either 4°C until physicochemical analysis or -80°C until DNA extraction.

### Measurements of soil edaphic properties

The edaphic properties of all root-associated soil samples were characterized at Hill Laboratories (Christchurch, New Zealand), where soil pH, Olsen phosphorus (mg/L), sulphate sulphur (mg/kg), total carbon (TC; %), organic matter (%), total nitrogen (TN; %), C:N ratio, potentially available N (kg/ha), anaerobically mineralizable N (AMN; %), AMN:TN ratio, and volume weight (g/mL) were determined using the protocols described by Wakelin et al. (2013).

### Plant DNA extraction and matK gene sequencing

The DNA of each PS was extracted using the DNeasy Plant Mini Kit (QIAGEN), utilizing cryogenic tissue grinding of plant leaves with a sterilized mortar and pestle in liquid nitrogen. For phylogenetic inference, the Maturase K gene (matK) was amplified using the primers MatK472F (5′-CCRTCATCTGGAAATCTTGGTT-3′) and MatK1248R (5′-GCTRTRATAATGAGAAAGATT TCTGC-3′) (Fatima et al. 2019). PCR conditions consisted of an initial denaturation step of 94°C for 5 min followed by 35 cycles of 94°C for 30 sec, 56°C for 30 sec, and 72°C for 42 sec, followed by a final extension at 72°C for 10 min. The PCR reaction mixture consisted of 1 × PCR buffer, 0.5 mmol L^−1^ dNTPs, 0.25 μmol L^−1^ of each primer, 1 U Taq polymerase, and 5–50 ng of template DNA. The PCR products were purified using the QIAquick PCR Purification Kit, and the purified DNA was sequenced using Sanger sequencing at Macrogen (Seoul, Korea). The quality of the sequencing data was checked and edited using Sequencer software version 5.4.6 (Genecodes Corp, Ann Arbor, MI, USA). MEGA X (Kumar et al. 2018) was then used for sequence alignment and phylogenetic analysis. Briefly, matK gene‐based sequences were aligned using MUSCLE, and overhanging nucleotides were removed and then re-aligned. Distance matrices and phylogenetic trees were constructed using the maximum likelihood method and the Tamura–Nei model (Tamura and Nei 1993).

### Soil DNA extraction and 16S rRNA gene/ITS region sequencing

Soil DNA was extracted from 0.25 g of soil using a DNeasy PowerSoil Kit (QIAGEN) according to the manufacturer’s protocol and quantified using a Nanodrop spectrophotometer. Subsequent Illumina amplicon sequencing followed the Earth Microbiome Project’s (EMP) protocol (Caporaso et al. 2012). In short, the bacterial 16S rRNA gene was amplified using the primers 515F (5′- GTGYCAGCMGCCGCGGTAA -3′) and 806R (5′- GGACTACNVGGGTWTCTAAT -3′) targeting the V4–V5 regions as described previously (Apprill et al. 2015, Parada et al. 2016). The fungal ITS region was amplified using the primers ITS1f (5′- CTTGGTCATTTAGAGGAAGTAA -3′) and ITS2 (5′- GCTGCGTTCTTCATCGATGC -3′) as described previously (Bokulich and Mills 2013, Hoggard et al. 2018). After PCR amplification, samples were purified using a Magnetic Bead PCR Cleanup Kit (GeneaidTM) and pooled in equimolar concentrations. The purified PCR products were used to prepare DNA libraries following the Illumina TruSeq DNA library preparation protocol using the Illumina MiSeq Reagent Kit v2. Illumina sequencing was performed at the Australian Genome Research Facility (Melbourne, Australia) using 2 × 150 bp pair-end chemistry on a MiSeq platform following the manufacturer’s guidelines.

### Statistical analysis

Following sequencing, paired-end fastQ files were processed into amplicon sequence variants (ASVs) using the DADA2 version 1.18 workflow (Callahan et al. 2016). Briefly, the forward and reverse reads were quality-filtered, trimmed, and denoised before being merged into ASVs. Chimeric ASVs were removed, and taxonomies were assigned to each ASV using the Ribosomal Database Project (RDP) Classifier (Wang et al. 2007) and the UNITE (Abarenkov et al. 2021) databases. Following DADA2 processing, ASV count tables were filtered to remove unidentified and unwanted phyla (i.e. Cyanobacteria/Chloroplasts) and singletons. The ASV count tables were rarefied to adjust for differences in library size between samples. Before rarefaction, samples with low read counts were removed to avoid excessive data loss. Rarefaction curves displaying the number of ASVs in each sample have been provided in Supplementary Figs S1 and S2. The number of replicates per PS that were included in the rarefied 16S (henceforth reported as ‘bacterial’) and ITS (henceforth reported as ‘fungal’) ASV datasets is displayed in Supplementary Table S1. In total, all the PS had at least three replicates in the rarefied fungal ASV dataset. In the rarefied bacterial ASV dataset, 35 out of the 37 PS had at least three replicates; however, only two replicates remained for the PS *Chionochloa conspicua* and *Trifolium repens* following rarefaction.

The rarefied bacterial and fungal ASV datasets were analysed separately using the multivariate statistical analyses outlined below. Maximum likelihood phylogenetic trees were built using FastTree2 (Price et al. 2010). To provide estimates of alpha diversity, Faith’s phylogenetic diversity (PD) and species richness (SR) were calculated for each sample in Picante R (Kembel et al. 2010). The PD index assesses the PD of a community and is defined as the sum of the total phylogenetic branch length separating taxa in a community (Faith 1992, Kembel et al. 2010). In contrast, the SR index calculates the total number of taxa in a community based on their identity alone—no phylogenetic information is factored into the calculation. The differences in the PD and SR index between plant host-related factors were tested for significance using Kruskal–Wallis tests and pairwise Wilcoxon tests with Bonferroni correction.

To estimate the phylogenetic distances in microbial community composition between samples, weighted UniFrac distances were calculated on rarefied bacterial and fungal ASV count tables (Lozupone et al. 2011). Differences in bacterial and fungal community composition between the plant-related factors were tested for significance using permutational multiple analysis of variance (PERMANOVA) on distance metrics using the adonis2 (by = ‘terms’) function in vegan R (Oksanen et al. 2019) and pairwiseAdonis (Martinez Arbizu 2020). The differences in community composition were visualized using non-metric multidimensional scaling (NMDS) ordination plots. To estimate the within-group variance amongst samples, the average distance of individual samples to the group centroid (beta dispersion) was calculated using the betadisper function in phyloseq R (McMurdie and Holmes 2013). Permutation tests were used to determine significant differences in the within-group variance between plant-related factors.

The weighted UniFrac distances were correlated to differences in soil physicochemical properties using Mantel tests. In addition, weighted UniFrac distances were correlated to matrices of matK sequence similarity using Mantel tests. MatK similarity matrices were constructed to represent the phylogenetic relatedness between the different PS under investigation. Observations of the phylogenetic tree generated from the matK phylogeny showed sensible grouping of PS to their taxonomic positioning. Hierarchical clustering analysis was performed on the weighted UniFrac distance matrices and matK distance matrices using the complete linkage method in Stats R. Following this, dendrograms were constructed to visually compare differences in the clustering patterns of PS based on the weighted UniFrac distances of their fungal and bacterial communities versus their matK distances in ape R (Paradis and Schliep 2019).

Variance partitioning (VP) analysis was performed in vegan R to partition the variance observed in bacterial and fungal community composition (as represented by weighted UniFrac distances) to the plant-related factors (Oksanen 2019). Four explanatory matrices were constructed to represent the different influencing factors. These were: PS; plant life history (PLH) (i.e. provenance + life span + functional group); plant rhizosphere traits (PRT) (i.e. mycorrhizal association + N_2_ fixation); and SC. All unexplained (residual) variation from VP analysis was tentatively assigned to represent the influence of stochastic processes. Following VP analysis, distance-based redundancy analysis (db-RDA) was performed to test the significance of each explanatory matrix whilst conditioning for the other three matrices. In addition, forward stepwise selection was performed to identify the soil physicochemical properties that best accounted for the community variance that was partitioned to the influence of SC. Pairwise differences in the soil physicochemical properties selected by the forward selection model between the 37 different PS were identified using pairwise *t*-tests with Holm correction.

The rarefied bacterial and fungal ASV tables were used as input for differential abundance analysis. First, the R package pime was used to select bacterial and fungal ASVs that best defined the microbiome of each PS (Luiz Fernando 2020). Prevalence intervals with an out-of-bag (OOB) error rate of 0% were selected as cut-offs. For fungal ASVs, this was a prevalence of 75%, which retained 217 ASVs and 1 168 389 sequences. For bacterial ASVs, this was a prevalence of 80%, which retained 771 ASVs and 433 795 sequences. PIME-filtered ASV count tables were used as input for differential abundance analysis using metagenomeSeq R (Paulson et al. 2013), where the log change estimate of each ASV between different PS was calculated using the fitLogNormal function. Significant differences in the log change estimates of ASVs between PS were determined using permutation tests (*n* = 999) with correction for multiple comparisons using the Holm–Bonferroni method (holm). Heatmaps were produced using pheatmap R (Kolde and Kolde 2018) to display (a) the bacterial and fungal ASVs with significant log change estimates across PS and (b) the correlation shared between different PS (Pearson’s) based on the log change estimates of their bacterial and fungal ASVs.

## Results

### Microbial species richness and phylogenetic diversity

There were no significant differences in the SR of bacterial communities across any of the plant-related factors (Table 1). However, the Faith’s diversity of bacterial communities was significantly higher in native versus exotic plants and in non-N_2_ fixing versus N_2_ fixing plants.

**Table 1.** The results of Kruskal–Wallis tests that were performed to identify significant differences in the SR and Faith’s diversity of root-associated bacterial and fungal ASVs across different plant-related factors.

	Bacterial ASVs						Fungal ASVs					
Factor	SR			Faith’s diversity			SR			Faith’s diversity		
	*H* value	*P* value	DF	*H* value	*P* value	DF	*H* value	*P* value	DF	*H* value	*P* value	DF
PS	44.16	0.17	36	49.94	0.06	36	54.02	0.03*	36	54.46	0.02*	36
Plant genus	35.26	0.23	30	41.12	0.08	30	47.28	0.02*	30	44.50	0.04*	30
Plant family	14.22	0.36	13	16.80	0.21	13	18.25	0.15	13	19.58	0.11	13
Plant order	13.59	0.26	11	15.03	0.18	11	17.67	0.09	11	19.17	0.06	11
Plant class	2.43	0.30	2	1.91	0.39	2	6.45	0.04*	2	4.64	0.10	2
Provenance^1^	1.50	0.22	1	5.89	0.02*	1	0.90	0.34	1	0.42	0.52	1
N_2_ fixing	2.13	0.14	1	4.96	0.03*	1	0.43	0.51	1	0.79	0.37	1
Life span^2^	1.34	0.51	2	2.18	0.34	2	6.28	0.04*	2	7.11	0.03*	2
Functional group^3^	2.25	0.52	3	2.42	0.49	3	6.68	0.08	3	8.60	0.04*	3
Mycorrhizal association^4^	0.41	0.81	2	2.44	0.29	2	4.37	0.11	2	6.27	0.04*	2

* *P* value < 0.05

1 exotic or native to Aotearoa New Zealand

2 annual, perennial, or long-lived

3 tree, forb, grass, or shrub

4 Arbuscular mycorrhizal fungi (AMF), ectomycorrhizal fungi (EMF), or no mycorrhizal association DF = degrees of freedom.

For fungal communities, both Faith’s diversity and SR were significantly higher in annual versus long-lived plants (Table 1). The PD of fungal communities was significantly lower in trees versus shrubs and grasses and significantly higher in non-mycorrhizal versus ectomycorrhizal plants. The mean (± SD) values for Faith's diversity and SR of fungal and bacterial across the different plant metadata factors can be seen through Supplementary Tables S2–S11.

### Microbial beta-diversity and community composition

Bacterial and fungal microbial community composition was significantly different between all plant-related factors (Table 2). PS and genus were the factors that reported the highest *R*^2^ values, thus accounting for most of the explained variation in bacterial and fungal community composition (Table 2; see also Supplementary Figs S3 and S4 in Supplementary Data). Although significant, the *R*^2^ values for many of the plant-related factors representing functional plant traits (i.e. provenance, functional group, primary mycorrhizal association, life span, and N_2_ fixation) were all low (*R*^2^ < 0.07). Bacterial and fungal communities both exhibited a heterogeneous dispersion and a high degree of within-group variability. The degree of beta-dispersion (‘*F* value’) observed in bacterial communities was significantly different across the following factors: PS, plant genus, plant family, plant order, and mycorrhizal association (Table 2). For fungal communities, significant beta-dispersion values were observed by plant family, plant order, plant class, provenance, and mycorrhizal association (Table 2).

**Table 2.** The results of PERMANOVA tests (*R*^2^ values) that were performed to identify significant differences in the composition of root-associated microbial communities across different plant-related factors.

	Bacterial ASVs		Fungal ASVs	
Factor	*R* ^2^	*F* value	*R* ^2^	*F* value
PS	0.342***	1.883**	0.301***	1.165
Plant genus	0.295***	2.263***	0.259***	1.388
Plant family	0.150***	3.182***	0.120***	3.858***
Plant order	0.138***	3.401**	0.107***	5.020***
Plant class	0.032***	2.531	0.020**	3.892*
Provenance	0.029***	0.688	0.023***	10.460**
N_2_ fixing	0.012**	0.961	0.018***	0.636
Life span	0.047***	2.654	0.022***	1.461
Functional group	0.069***	1.057	0.041***	0.764
Mycorrhizal association	0.048***	7.906**	0.030***	3.299*

* *P* value < 0.05; ** *P* value < 0.01; *** *P* value < 0.001

PERMANOVA tests were performed on weighted UniFrac distance matrices, which were calculated using bacterial and fungal ASV tables. The results of permutation-based tests of beta-dispersion are displayed (*F* values), were performed to identify significant differences in the within-group variance of bacterial and fungal communities for each plant-related factor.

### MatK gene sequence similarity

The distances in matK gene sequence similarity between the different PS did not significantly correlate to the corresponding weighted UniFrac distances for their bacterial (Mantel *r* = 0.134, *P* value = 0.075) or fungal (Mantel *r* = 0.040, *P* value = 0.306) communities. This is illustrated in Supplementary Figs S5 and S6, as the hierarchical clustering patterns of the different PS based on their matK gene sequences versus their bacterial and fungal community composition had little correspondence.

### Variance partitioning (VP) analysis

**Figure 1.**
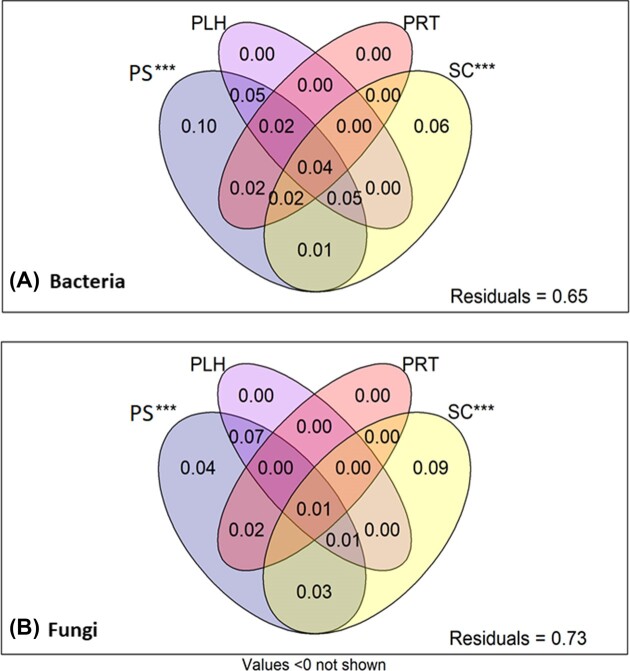
The proportion of explained variance attributed to each explanatory matrix, as identified using VP analysis. VP analysis was performed on weighted UniFrac distances of (A) bacterial ASVs and (B) fungal ASVs. Explanatory matrices used as input were PS, PLH, PRT, and SC. The significance of each explanatory matrix in accounting for community variation is displayed, whereby *** denotes *P* value < 0.001.

VP analysis identified that, cumulatively, PS, PLH, PRT, and SC explained 34.59% of bacterial community variance and 27.27% of fungal community variance (Fig. 1). Thus, both bacterial and fungal communities exhibited a high degree of residual, unexplained variation (65.41% and 72.73%, respectively). When individual explanatory matrices were tested for significance using partial db-RDA, both plant identity (Bacteria: *F* value = 1.40, *P* value < 0.001, Fungi: *F* value = 1.32, *P* value < 0.001) and SC (Bacteria: *F* value = 1.39, *P* value < 0.001, Fungi: *F* value = 1.41, *P* value < 0.001) significantly accounted for community variance.

When the other explanatory matrices were conditioned out of the model, plant identity alone accounted for 9.52% of bacterial community variance and 3.65% of fungal community variance. In contrast, SC accounted for 5.67% of bacterial community variance and 9.40% of fungal community variance. PLH (Bacteria: *F* value = 0.00, *P* value > 0.05, Fungi: *F* value = 0.00, *P* value > 0.05) and PRT (Bacteria: *F* value = 0.00, *P* value > 0.05, Fungi: *F* value = 0.00, *P* value > 0.05) did not significantly account for any bacterial or fungal community variation.

**Figure 2.**
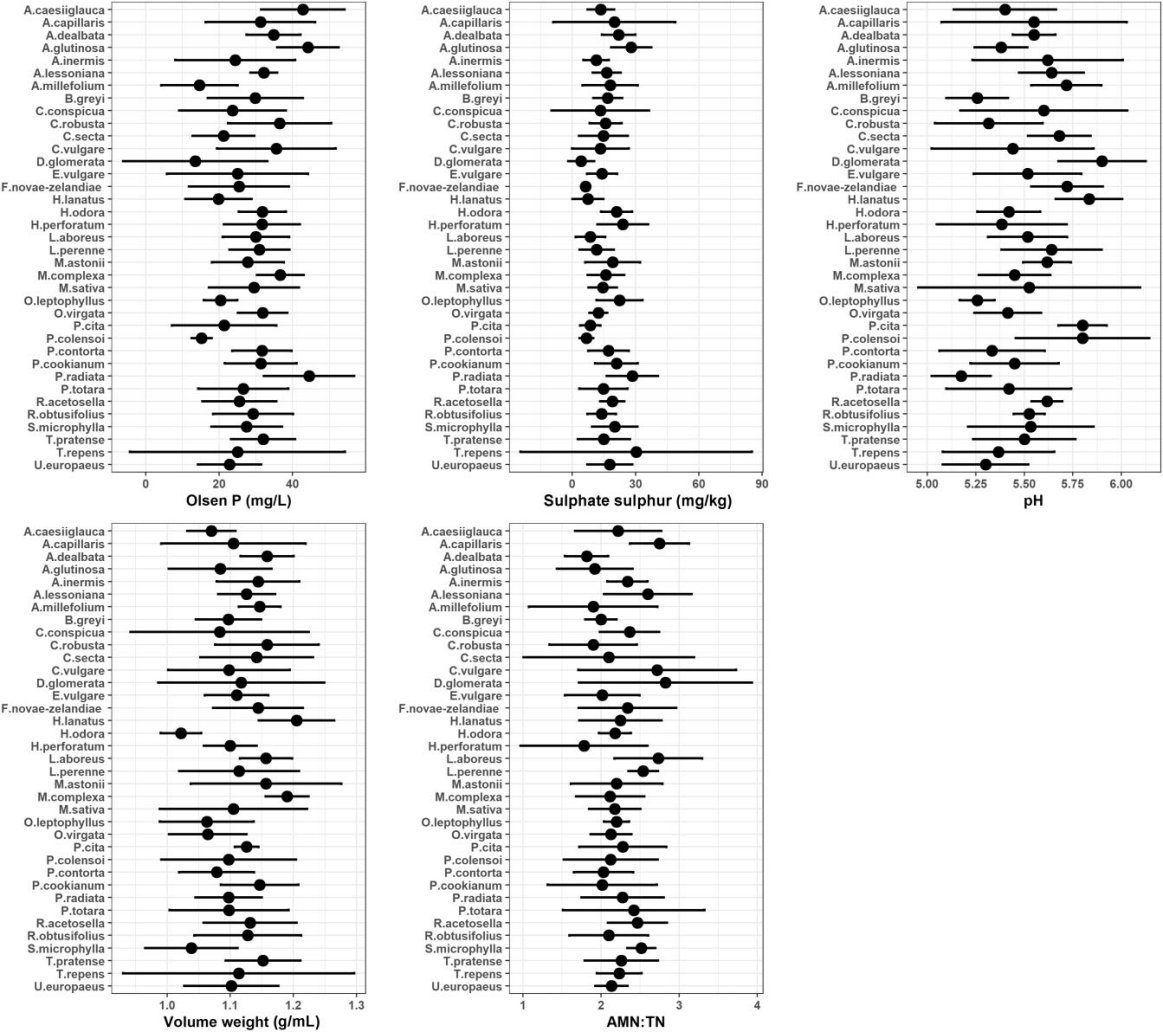
The mean ± SE values of the soil properties that were identified by forward selection models to significantly account for the variation in root-associated soil microbial communities. The soil properties Olsen P (mg/L), sulphate sulphur (mg/kg), and pH significantly accounted for bacterial community variation, whilst Olsen P (mg/L), AMN:TN ratio, and volume weight (g/mL) significantly accounted for fungal community variation.

The composition of the root-associated soil microbiome may be indirectly influenced by plant-induced modification of the physicochemical environment. When looking at soil physicochemical properties that best accounted for bacterial community variation, forward selection models identified Olsen P (*F* value = 10.76, *P* value < 0.05), sulphate sulphur (*F* value = 2.54, *P* value < 0.05), and pH (*F* value = 4.54, *P* value < 0.05) to be significant. For fungal communities, forward selection models identified Olsen P (*F* value = 9.95, *P* value < 0.05), AMN:TN (*F* value = 3.69, *P* value < 0.01), and volume weight (*F* value = 2.85, *P* value < 0.05) to be significant. The values for these soil properties were variable between the 37 different PS (Fig. 2). Pairwise *t*-tests identified that Olsen P was significantly higher (*P* adjusted < 0.05) in *Acaena caesiiglauca* (vs. *Achillea millefolium, Dactylis glomerata*, and *Poa colensoi*), *Alnus glutinosa* (vs. *Ach. millefolium, D. glomerata, Holcus lanatus, Ozothamnus leptophyllus*, and *P. colensoi*), and *Pinus radiata* (vs. *Ach. millefolium, D. glomerata*, and *Po. colensoi*). Volume weight was significantly higher in *Ho. lanatus* (vs. *Hebe odora, O. leptophyllus, Olearia virgata*, and *Sophora microphylla*) and *Muehlenbeckia complexa* (vs. *He. odora* and *S. microphylla*). Soil pH was significantly higher in *D. glomerata* and *Ho. lanatus* (vs. *O. leptophyllus, Pi. contorta, Pi. radiata, Brachyglottis greyi, Coprosma robusta*, and *Ulex europaeus*), *Ach. millefolium* (vs. *Pi. radiata*), and *Po. colensoi* and *P. cita* (vs. *Pi. radiata, O. leptophyllus*, and *B. greyi*). Although forward selection models identified AMN:TN and sulphate sulphur to significantly influence fungal and bacterial community composition, no significant pairwise differences were determined between the 37 PS for these properties. The mean ± SD values for all soil physicochemical properties associated with each PS are presented in Supplementary Table S12.

### Taxonomic differentiation across plant species

**Figure 3.**
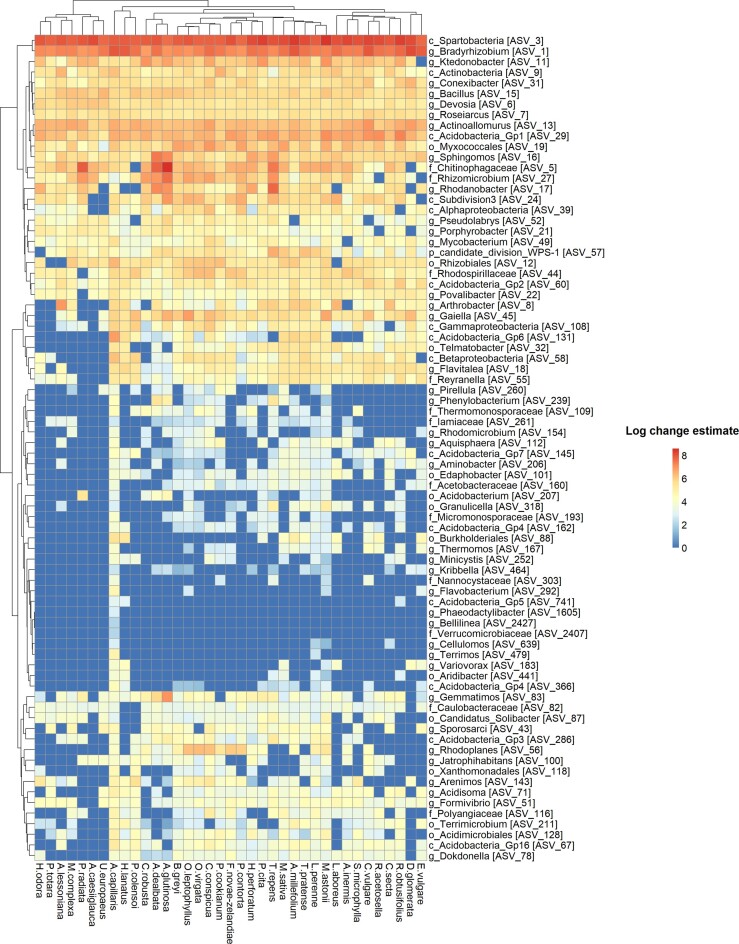
The log change estimates of the 78 bacterial ASVs that were identified to have significant differential abundance values (*P* adjusted < 0.05) across the PS under investigation. The taxonomic identity of each bacterial ASV is presented, with each bacterial ASV represented by the most refined taxonomic rank that could be accurately assigned.

**Figure 4.**
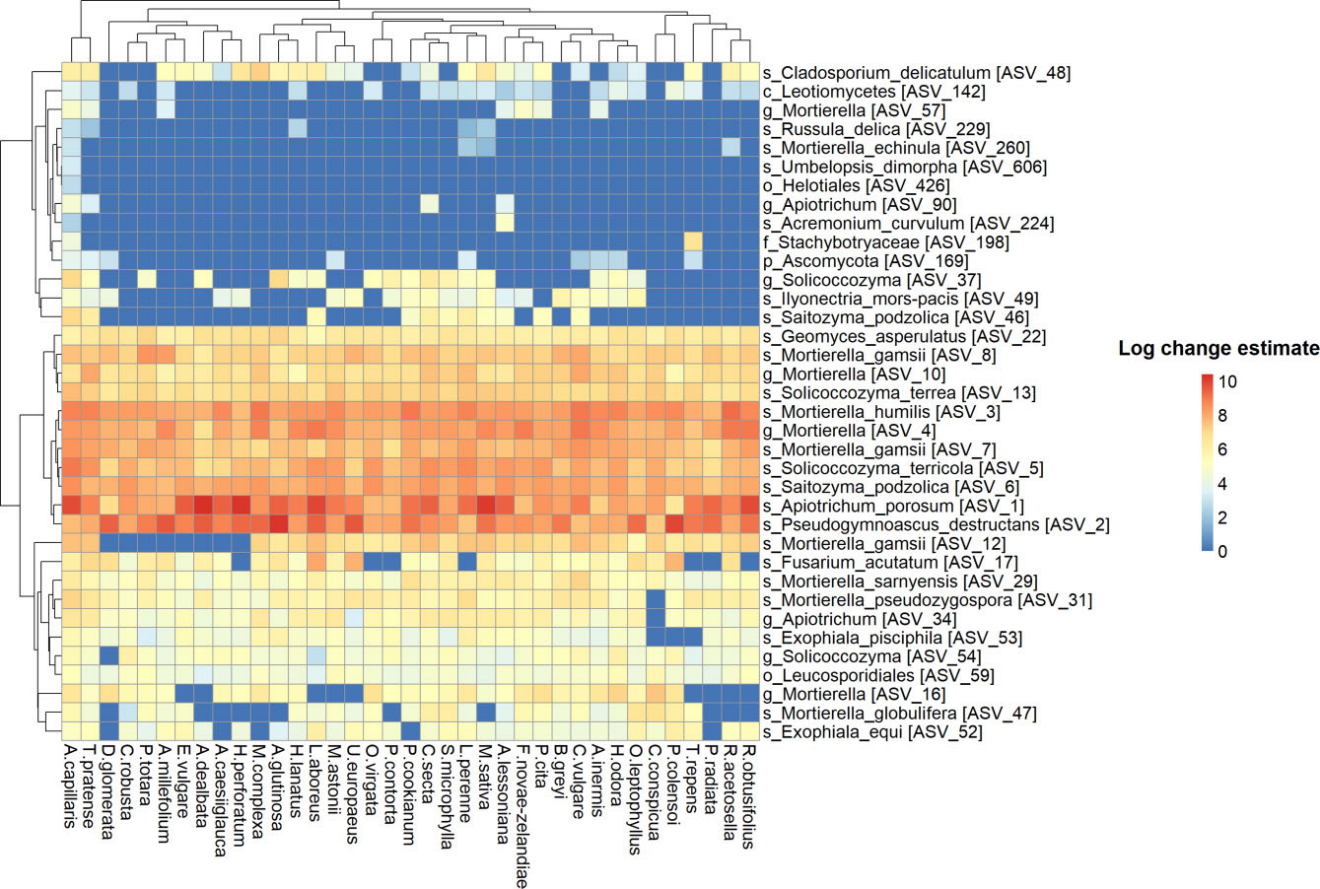
The log change estimates of the 36 fungal ASVs that were identified to have significant differential abundance values (*P* adjusted < 0.05) across the PS under investigation. The taxonomic identity of each fungal ASV is presented, with each fungal ASV represented by the most refined taxonomic rank that could be accurately assigned.

Out of the 771 bacterial ASVs that were retained following PIME filtering and used as input for differential abundance analysis, only 10.12% (78 ASVs) were identified to be differentially abundant amongst PS (*P* adjusted < 0.05). Furthermore, out of the 217 fungal ASVs retained following PIME filtering, only 16.59% (36 ASVs) were differentially abundant amongst PS (*P* adjusted < 0.05). Figures 3 and 4 display the bacterial and fungal ASVs that had significantly different log change estimates across the PS under investigation. These results highlight that there were no large patterns of taxonomic differentiation amongst PS, that is, PS did not have markedly distinct taxonomic compositions. One exception was *Agrostis capillaris* (common bent or brown top grass), whose bacterial and fungal taxa were more evidently differentiated compared to the other PS. All the PS shared a significant (*P* adjusted < 0.05) positive correlation based on the log change estimates of their bacterial (Pearson’s r correlation; 0.70 ± 0.08 SD) and fungal ASVs (Pearson’s r correlation; 0.69 ± 0.08 SD). These results indicate a low divergence of PS based on their root-associated soil microbiome (Supplementary Figs S7 and S8).

## Discussion

The root-associated soil microbiome provides fundamental roles in supporting plant health, productivity, and resilience against abiotic and biotic stressors (Mendes et al. 2011, Berendsen et al. 2012, Penton et al. 2014). Thus, pinpointing how different components of the plant-root-soil interface drive microbial selection and establishment is key for us to manage plant and soil health into the future. However, identifying the primary processes that drive microbial assembly is complex and is suggested to be by the interacting influences of plant genotype, developmental stage, root exudates, root morphology, PLH, soil type, and previous soil history (Chaparro et al. 2014, Zhao et al. 2019, Zhou et al. 2020, Cordovez et al. 2021). By controlling for the starting soil mixture and surrounding environmental conditions, our research aimed to identify how the different phylogenetic, functional, and ecological traits of PS were related to the assembly of their root-associated soil microbiome.

### The phylogenetic relatedness of plant hosts shared no relationship to the similarity in their root-associated soil microbiome

Our research aimed to test whether the phylogenetic relatedness of PS was correlated with the phylogenetic similarity of their root-associated soil microbiome—a hypothesis that has been supported by previous research (Bouffaud et al. 2014, Lambais et al. 2014, Yeoh et al. 2017, Lei et al. 2019, Kaplan et al. 2020, Hartman et al. 2023). Our results did not support this hypothesis, as the phylogenetic similarity in root-associated soil microbiomes did not correlate with the phylogenetic similarity between different PS. With the increase in phylogenetic ranking from PS to class level, we observed a consistent decline in the degree of microbial community variation that could be accounted for by plant phylogenetic origin. That is, higher phylogenetic rankings such as plant class and order only explained a small amount of compositional variation compared to PS-level identity. This suggests that, whilst PS may be used as a predictor of the root-associated soil microbiome, higher taxonomic rankings of PS cannot. Similar findings were observed by Fitzpatrick et al. (2018), who identified that although PS identity was a significant factor shaping rhizosphere assembly, the emergent structure of the rhizosphere microbiome shared no relationship with the phylogenetic relatedness between plant hosts.

### Plant species and plant-induced changes in soil physicochemistry were the strongest predictors of microbial assembly

Although patterns in microbial assembly did not relate to the phylogenetic relationships among PS, species identity and differences in SC were the two most significant factors that accounted for bacterial and fungal community variation—a finding also observed by Burns et al. (2015). Whilst both factions were significant, PS identity accounted for a greater proportion of bacterial community variance than SC. Several studies have reported PS identity to be a significant factor in shaping microbial assembly and community structure (Garbeva et al. 2008, Berg and Smalla 2009, Becklin et al. 2012, Burns et al. 2015). These plant-species-dependent effects on microbial assembly have been attributed to the release of carbon-rich root exudates, which selectively enrich and recruit specific root-associated soil microorganisms (Bais et al. 2006), with the quality and composition of root exudates varying according to PS and plant developmental stage (Badri and Vivanco 2009, Zhalnina et al. 2018).

For fungal communities, SC was identified to account for a higher amount of community variance than PS identity. In our experiment, all PS were planted in the same starting soil mixture. As such, these effects are not associated with differences in soil type or edaphic properties per se but are changes that the plants themselves have directly expressed on the rhizosphere and soil environment. Furthermore, plant-driven changes in the composition of root-associated microorganisms throughout the early stages of microbial assembly may also have indirectly driven the shifts observed in SC. Plants can directly modify the conditions of their surrounding soil physicochemical environment via nutrient uptake/loss or by the chemical signatures of their leaf litter, roots, and root exudates. Plants can also shape their soil physicochemical environment indirectly by driving changes in the activity and composition of their root-associated microorganisms (Rengel and Marschner 2005, Waring et al. 2015, Henneron et al. 2020). Root-associated microorganisms have key roles in the transformation and mobilization of inorganic and organic substrates into more plant-accessible soil nutrients, meaning that they can have a transformative impact on soil nutrient cycling (Finzi et al. 2015, Dlamini et al. 2022; Dotaniya and Meena 2015). Plant-induced changes in SC provide an example of how secondary deterministic processes can indirectly shape microbial assembly. As plant roots modify the conditions of the rhizosphere and root-associated soil environment, this encourages the growth of microorganisms that can occupy the modified habitat space (Hinsinger 2001, Liang et al. 2002, Bell et al. 2015, van Veelen et al. 2020, Hernández-Cáceres et al. 2022).

Plant-available P, such as that measured by Olsen P (bicarbonate extractable), is a key measure of soil fertility and ecosystem productivity (Vitousek et al. 2010). In our research, Olsen P had a particularly strong relationship with changes in root-associated soil fungal and bacterial communities. In particular, the root-associated soils from the PS *Pi. radiata, Al. glutinosa*, and *Ac. caesiiglauca* had high Olsen P values compared to the other PS. These observations may demonstrate the process of plants mobilizing soil nutrients essential for their individual growth and fitness (Will 1978, Chen et al. 2002, Tallec et al. 2009, Varin et al. 2009) and how these are linked to changes in soil microbiology. When root-induced changes in soil chemistry influence microbial assembly, this ultimately impacts plant health and performance, and thereby success in the ecosystem. These form plant-soil feedback mechanisms that amplify over successive life cycles (van der Putten et al. 2013, Bennett and Klironomos 2019) and are profoundly connected with ecosystem-level processes.

### The functional traits of plant species did not influence microbial community assembly

In our study, plant functional traits such as life span, functional group, provenance, N_2_ fixation, and mycorrhizal association were not identified as strong drivers in microbial community assembly. It is important to consider that we examined root-associated soil microbes, but not microbes that colonize and develop symbiotic relationships with plant roots such as endophytes or mycorrhizal fungi. Had we examined the assembly patterns of plant symbiotic microbes and not free-living soil microbes, we may have observed the functional traits of host plants to have had a more pronounced impact on patterns of microbial assembly. Our findings are complementary to Hartman et al. (2023), who identified that the symbiotic associations of plant hosts significantly impact the root microbiome. Unlike Hartman et al. (2023) and Bodenhausen et al. (2019), who sampled the root-associated microbiome, our study examined soil adjacent to plant roots. Thus, discrepancies between the findings of our research and Hartman et al. (2023) are due to the clearly different sampling methodologies, as we sampled soils at a greater physical distance from the root. Additionally, our research investigated root-associated soil microbial assembly following (a) a single life cycle of the plant and (b) at a single time point during the plant’s developmental stage. Thus, the absence of clear divergences in microbial assembly between plants with contrasting functional traits may be a consequence of our experiment’s relatively short duration or other factors. For example, although we studied plants with different life cycle strategies (i.e. annual vs. perennial), we did not study them over repeated life cycles, where the outcomes of their contrasting life histories may have modified their soil environment to a degree that influenced microbial assembly. Several studies have reported the soil microbiome to shift according to plant development, influenced by changes in plant root morphology and exudate release with each developmental stage (Micallef et al. 2009, Chaparro et al. 2014). Furthermore, the divergence in microbial assembly between PS may amplify over successive life cycles (Cordovez et al. 2021). This increasing divergence is driven by plant-soil feedback mechanisms, whereby successive modifications in soil biotic and abiotic conditions by plants exert greater selection pressures on their root-associated soil microbiota (Hu et al. 2018).

### Root-associated soil microbiomes exhibited a large degree of unexplained variation

Niche-based theories of microbial community assembly assert that deterministic processes govern community structure, such as adaptive species traits, biotic interactions, and environmental filtering (Dini-Andreote et al. 2015, Zhou and Ning 2017). As discussed, aside from plant identity and plant-induced changes in SC, the functional plant traits measured in our study accounted for very little of the variation observed in root-associated soil microbial communities. Our results identified that a large amount of the compositional variation in root-associated soil communities remained unexplained, with over 73% of fungal community variation and 65% of bacterial community variation unaccounted. Given the breadth of variables we assessed, much of this variation may represent elements of stochastic processes driving random community assemblage. More recently, there has been a growing body of literature recognizing the degree to which stochastic processes may govern the resulting structure of microbial communities (Caruso et al. 2011, Zhang et al. 2016, Zhou and Ning 2017, Chen et al. 2019, Hou et al. 2021, Huang et al. 2022).

The PS under investigation in this study were at relatively early stages of succession and growth (plants were grown for 10 months), which may explain the large amount of unexplained compositional variation we observed. Stochastic processes are reported to dominate microbial assembly during the early stages of community establishment, as the roots of plant seedlings release an abundant supply of exudates, which reduces competitive biotic interactions (Dini-Andreote et al. 2015). However, throughout community development, microbiomes transition from random community assembly to more highly structured, niche-differentiated assemblages because of functional adaptations to the environmental selection pressures (Aguilar and Sommaruga 2020, Hu et al. 2020). As plants develop, they alter the bioavailability of resources according to their needs; thus, deterministic processes increasingly dominate microbial community assembly as the surrounding environment is increasingly modified by plant growth (Dini-Andreote et al. 2015). The modification of soil physicochemical properties by PS was observed for several of the PS in our study, with *Pi. radiata, Ac. caesiiglauca*, and *Al. glutinosa* driving changes in Olsen P, for example. It is possible that if the microbial communities were measured over longer periods, community assembly would be more evidently niche-differentiated as each plant exerted unique selection pressures within the root-associated soil environment.

## Conclusion

Our research findings identified that during the early stages of plant growth and establishment, PS identity and plant-induced changes in SC were the most significant factors that shaped root-associated soil microbial assembly. The functional traits of the PS under investigation, such as their life span, provenance, growth form, and mycorrhizal associations, did not significantly account for any of the structural variation observed in bacterial or fungal communities between plants. Although PS identity was determined to be a significant factor driving microbial assembly, the phylogenetic relationships shared between the 37 PS under investigation shared no relationship to the similarity of their root-associated soil microbiomes. Thus, our findings reject the hypothesis that plant phylogenetic relatedness can be used to predict the emergent structure of the root-associated soil microbiome.

## Supplementary Material

fiad126_Supplemental_File

## Data Availability

The R scripting code used for the data analysis presented in this research can be found at https://github.com/akbyers/rhizosphere_microbiome_assemblage
