# Supplementary material for: Plant species identity and plant-induced changes in soil physicochemistry—but not plant phylogeny or functional traits - shape the assembly of the root-associated soil microbiome
Source: FEMS Microbiol Ecol. 2023 Oct 10;99(11):fiad126. doi: 10.1093/femsec/fiad126 (PMC10589101; doi:10.1093/femsec/fiad126)
Supplement: fiad126_Supplemental_File [file fiad126_supplemental_file.docx]

**Supplementary Data**

**Plant species identity and plant-induced changes in soil physicochemistry – but not plant phylogeny or functional traits -** **shape the assembly of the root-associated soil microbiome.**

**Alexa-Kate Byers^1*^, Leo M. Condron^1^, Maureen O’Callaghan^2^, Lauren Waller^3^, Ian A. Dickie^4^, Steve A. Wakelin^5^**

^1^Bioprotection Aotearoa, Lincoln University, P.O. Box 85084, Lincoln 7647, New Zealand

^2^AgResearch Ltd, 1365 Springs Road, Lincoln 7674, New Zealand

^3^Ministry for Primary Industries, 34-38 Bowen Street, PO Box 2526, Wellington 6140, New Zealand

^4^Bioprotection Aotearoa, School of Biological Sciences, University of Canterbury, P.O. Box 4800, Christchurch 8140, New Zealand

^5^Scion Research Ltd, 10 Kyle Street, Riccarton, Christchurch 8011, Canterbury, New Zealand

*** Correspondence:**Corresponding Author [alexa.byers@lincoln.ac.nz](mailto:alexa.byers@lincoln.ac.nz)

**Table S1. The 37 different plant species investigated in this study ranged across different phylogenetic origins, life spans, provenances, functional groups, mycorrhizal associations, and nitrogen (N_2_) fixing forms. The number of replicates (n) included in the final bacterial and fungal ASV datasets is displayed.**

| **Plant species** | **Phylogenetic origin** | **Life span** | **Provenance** | **Functional group** | **Mycorrhizal association** | **N_2_ fixation** | **n: 16S dataset** | **n: ITS dataset** |
| --- | --- | --- | --- | --- | --- | --- | --- | --- |
| *Acacia dealbata* | Magnoliopsida; Fabales; Fabaceae | Perennial | Exotic | Tree | AMF | Yes | 6 | 6 |
| *Acaena caesiiglauca* | Magnoliopsida; Rosales; Rosaceae | Perennial | Native | Forb | AMF | No | 5 | 5 |
| *Acaena inermis* | Magnoliopsida; Rosales; Rosaceae | Perennial | Native | Forb | AMF | No | 4 | 5 |
| *Achillea millefolium* | Magnoliopsida; Asterales; Asteraceae | Perennial | Exotic | Forb | AMF | No | 5 | 6 |
| *Agrostis capillaris* | Magnoliopsida; Poales; Poaceae | Perennial | Exotic | Grass | AMF | No | 3 | 4 |
| *Alnus glutinosa* | Magnoliopsida; Fagales; Betulaceae | Long-lived | Exotic | Tree | EMF | Yes | 5 | 6 |
| *Anemanthele lessoniana* | Magnoliopsida; Poales; Poaceae | Perennial | Native | Grass | AMF | No | 4 | 5 |
| *Brachyglottis greyi* | Magnoliopsida; Asterales; Asteraceae | Perennial | Native | Forb | AMF | No | 6 | 7 |
| *Carex secta* | Magnoliopsida; Poales; Cyperaceae | Perennial | Native | Grass | No | No | 5 | 6 |
| *Chionochloa conspicua* | Magnoliopsida; Poales; Poaceae | Perennial | Native | Grass | AMF | No | 2 | 3 |
| *Cirsium vulgare* | Magnoliopsida; Asterales; Asteraceae | Annual | Exotic | Forb | AMF | No | 5 | 7 |
| *Coprosma robusta* | Magnoliopsida; Gentianales; Rubiaceae | Perennial | Native | Shrub | AMF | No | 6 | 5 |
| *Dactylis glomerata* | Magnoliopsida; Poales; Poaceae | Perennial | Exotic | Grass | AMF | No | 3 | 4 |
| *Echium vulgare* | Magnoliopsida; Boraginales; Boraginaceae | Annual | Exotic | Forb | No | No | 5 | 6 |
| *Festuca novae-zealandiae* | Magnoliopsida; Poales; Poaceae | Perennial | Native | Grass | AMF | No | 5 | 5 |
| *Hebe odora* | Magnoliopsida; Lamiales; Plantaginaceae | Perennial | Native | Shrub | AMF | No | 5 | 5 |
| *Holcus lanatus* | Magnoliopsida; Poales; Poaceae | Perennial | Exotic | Grass | AMF | No | 5 | 6 |
| *Hypericum perforatum* | Magnoliopsida; Malpighiales; Hypericaceae | Perennial | Exotic | Forb | AMF | No | 6 | 6 |
| *Lolium perenne* | Magnoliopsida; Poales; Poaceae | Perennial | Exotic | Grass | AMF | No | 5 | 5 |
| *Lupinus arboreus* | Magnoliopsida; Fabales; Fabaceae | Perennial | Exotic | Shrub | No | Yes | 5 | 7 |
| *Medicago sativa* | Magnoliopsida; Fabales; Fabaceae | Perennial | Exotic | Forb | AMF | Yes | 4 | 5 |
| *Muehlenbeckia astonii* | Magnoliopsida; Caryophyllales; Polygonaceae | Perennial | Native | Shrub | No | No | 6 | 6 |
| *Muehlenbeckia complexa* | Magnoliopsida; Caryophyllales; Polygonaceae | Perennial | Native | Shrub | No | No | 5 | 6 |
| *Olearia virgate* | Magnoliopsida; Asterales; Asteraceae | Perennial | Native | Shrub | AMF | No | 6 | 7 |
| *Ozothamnus leptophyllus* | Magnoliopsida; Asterales; Asteraceae | Perennial | Native | Forb | AMF | No | 6 | 7 |
| *Phormium cookianum* | Liliopsida; Asparagales; Asphodelaceae | Perennial | Native | Forb | AMF | No | 4 | 6 |
| *Pinus contorta* | Pinopsida; Pinales; Pinaceae | Long-lived | Exotic | Tree | EMF | No | 7 | 9 |
| *Pinus radiata* | Pinopsida; Pinales; Pinaceae | Long-lived | Exotic | Tree | EMF | No | 4 | 4 |
| *Poa cita* | Magnoliopsida; Poales; Poaceae | Perennial | Native | Grass | AMF | No | 5 | 6 |
| *Poa colensoi* | Magnoliopsida; Poales; Poaceae | Perennial | Native | Grass | AMF | No | 4 | 4 |
| *Podocarpus totara* | Pinopsida; Pinales; Podocarpaceae | Long-lived | Native | Tree | AMF | No | 5 | 5 |
| *Rumex acetosella* | Magnoliopsida; Caryophyllales; Polygonaceae | Perennial | Exotic | Forb | No | No | 5 | 3 |
| *Rumex obtusifolius* | Magnoliopsida; Caryophyllales; Polygonaceae | Perennial | Exotic | Forb | No | No | 3 | 4 |
| *Sophora microphylla* | Magnoliopsida; Fabales; Fabaceae | Perennial | Native | Shrub | AMF | Yes | 6 | 6 |
| *Trifolium pratense* | Magnoliopsida; Fabales; Fabaceae | Perennial | Exotic | Forb | AMF | Yes | 4 | 6 |
| *Trifolium repens* | Magnoliopsida; Fabales; Fabaceae | Perennial | Exotic | Forb | AMF | Yes | 2 | 3 |
| *Ulex europaeus* | Magnoliopsida; Fabales; Fabaceae | Perennial | Exotic | Shrub | AMF | Yes | 5 | 5 |


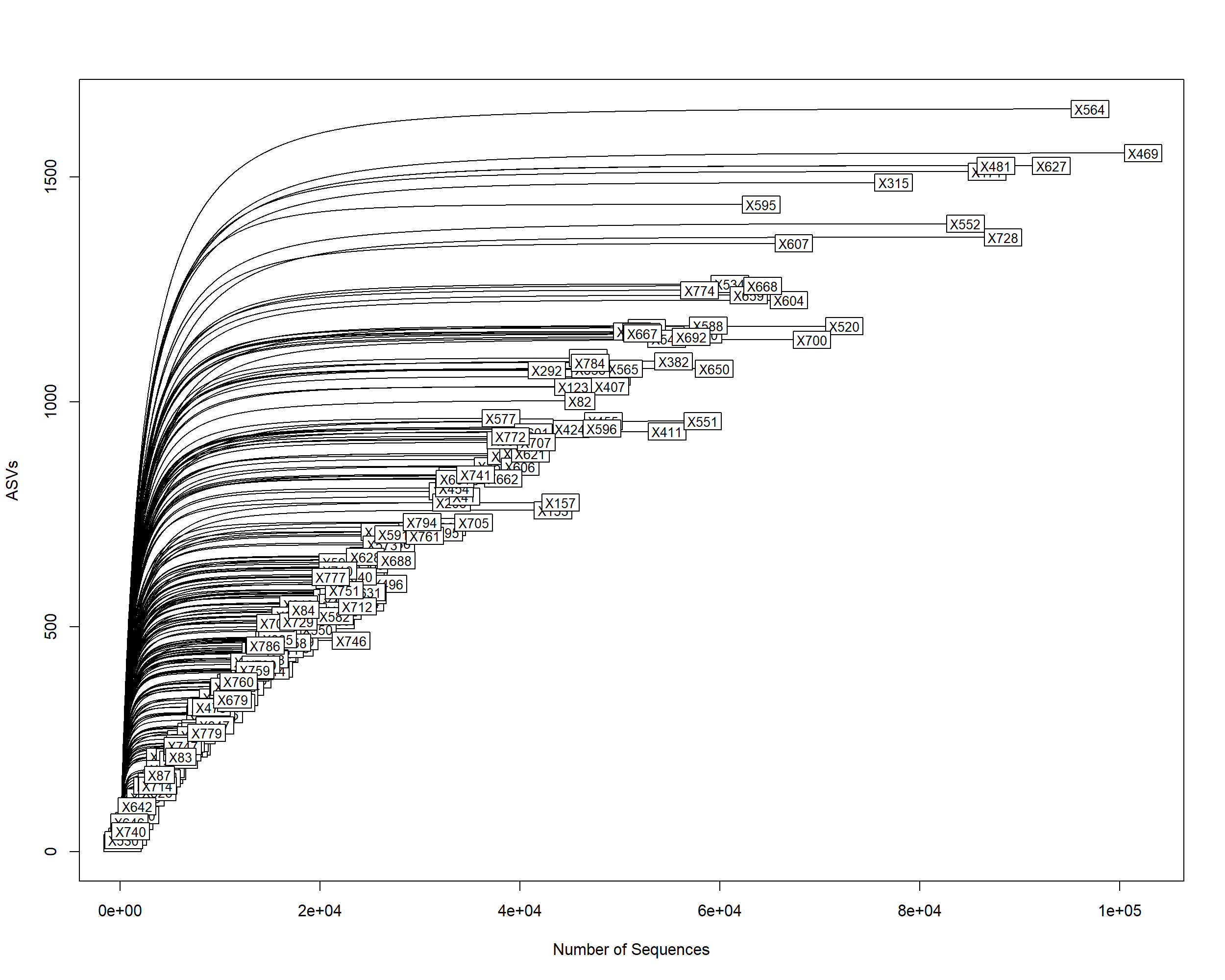


Figure S1. Rarefaction curves displaying the total number of bacterial ASVs per sample. The 22 samples that were removed from the bacterial ASV dataset before rarefaction were "128", "530", "183", "646", "740", "117", "642", "580", "23", "513", "625", "156", "669", "626", "714", "586", "87", "641", "226", "549", "120", and "453".


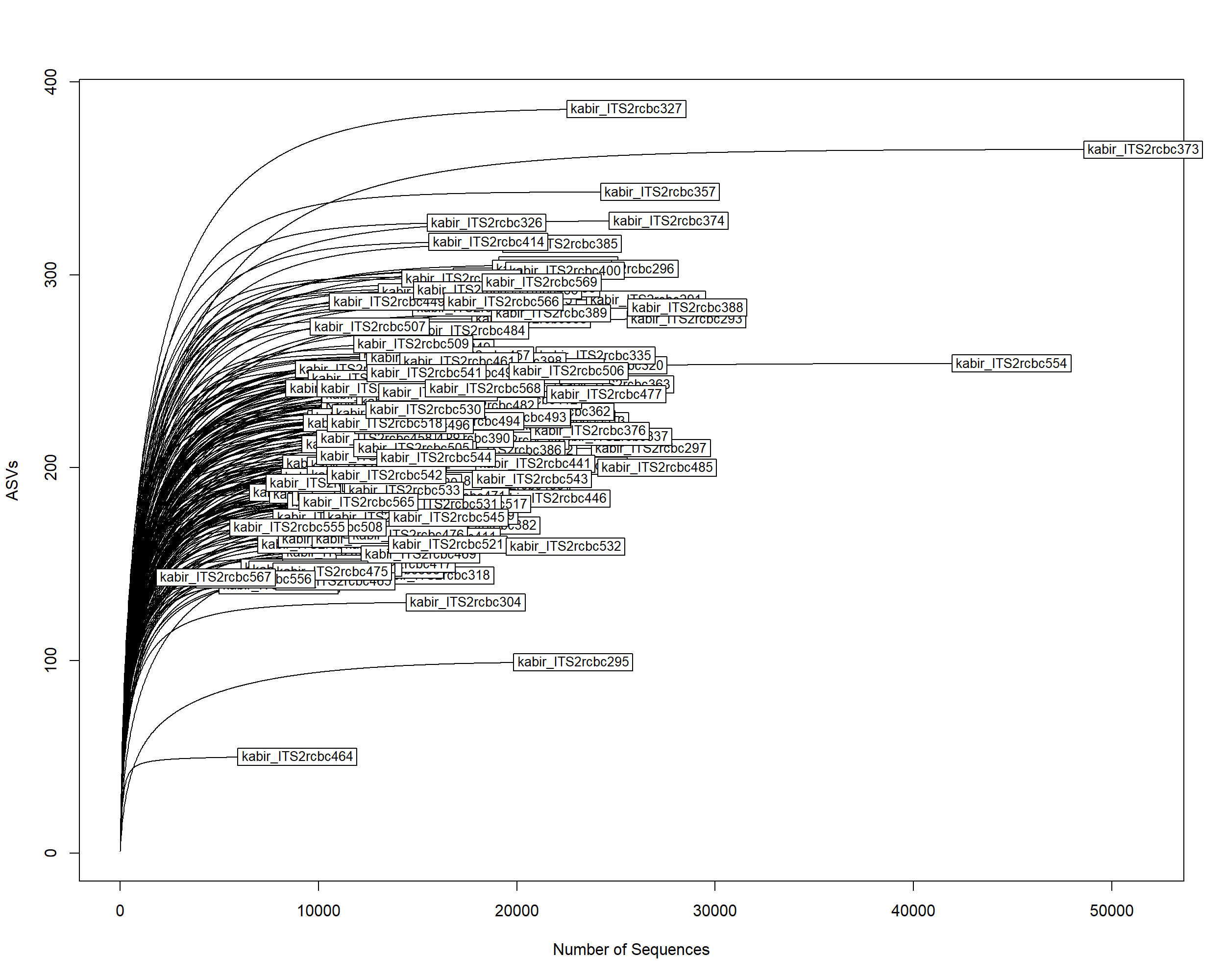


Figure S2. Rarefaction curves displaying the total number of fungal ASVs per sample. The six samples that were removed from the fungal ASV dataset before rarefaction were "kabir_ITS2rcbc567", "kabir_ITS2rcbc556", "kabir_ITS2rcbc459", "kabir_ITS2rcbc403", "kabir_ITS2rcbc555", and "kabir_ITS2rcbc464".

**Table S2. The mean ± SD Faith's diversity and species richness of fungal and bacterial soil communities are summarized by the species-level identity of their respective plant host.**

| **Plant species** | **Bacterial communities** | | **Fungal communities** | |
| --- | --- | --- | --- | --- |
|  | **Faith's diversity** | **Species richness** | **Faith's diversity** | **Species richness** |
| *A. dealbata* | 31.08 ± 7.2 | 502 ± 230.54 | 40.08 ± 4.81 | 191.17 ± 24.41 |
| *A. caesiiglauca* | 29.38 ± 10.04 | 421.6 ± 298.18 | 45.39 ± 10.62 | 223.6 ± 48.43 |
| *A. inermis* | 35.23 ± 5.95 | 508 ± 138.38 | 41.85 ± 7.67 | 188.6 ± 34.31 |
| *A. millefolium* | 37.43 ± 5.04 | 631 ± 186.16 | 44.03 ± 6.67 | 212.83 ± 34.52 |
| *A. capillaris* | 34.34 ± 7.11 | 475.33 ± 133.87 | 52.88 ± 7.99 | 237 ± 40.27 |
| *A. glutinosa* | 37.59 ± 4.99 | 683.4 ± 178.42 | 43.63 ± 6.93 | 212.17 ± 32.62 |
| *A. lessoniana* | 33.61 ± 12.89 | 543 ± 319.74 | 49.69 ± 4.13 | 244.6 ± 21.33 |
| *B. greyi* | 42.02 ± 5.35 | 730.67 ± 263.03 | 42.71 ± 6.39 | 199 ± 27.59 |
| *C. secta* | 33.51 ± 7.92 | 553.6 ± 245.82 | 45.95 ± 4.56 | 212.17 ± 31.68 |
| *C. conspicua* | 39.2 ± 4.71 | 521 ± 115.97 | 41.29 ± 5.88 | 178.33 ± 24.11 |
| *C. vulgare* | 32.5 ± 6.16 | 479 ± 121.53 | 47.92 ± 5.89 | 223.71 ± 29.49 |
| *C. robusta* | 39.24 ± 5.13 | 640.67 ± 176.79 | 39.74 ± 10.42 | 188 ± 49.2 |
| *D. glomerata* | 30.89 ± 10.57 | 448 ± 280.33 | 44.28 ± 10.75 | 216.75 ± 55.33 |
| *E. vulgare* | 35.6 ± 11.55 | 671.2 ± 411.52 | 51.75 ± 8.48 | 244.5 ± 48.94 |
| *F. novae-zelandiae* | 42.77 ± 9.37 | 767.8 ± 335.18 | 43.5 ± 6.39 | 203 ± 27.68 |
| *H. odora* | 37.61 ± 12.43 | 608.8 ± 397.97 | 51.05 ± 5.69 | 239.2 ± 26.32 |
| *H. lanatus* | 38.26 ± 12.8 | 676 ± 347.42 | 54.2 ± 8.86 | 260.83 ± 45.16 |
| *H. perforatum* | 38.16 ± 7.81 | 699.33 ± 247.23 | 45.37 ± 9.82 | 220.17 ± 52.57 |
| *L. perenne* | 47.32 ± 8.02 | 963 ± 268.71 | 49.7 ± 6.21 | 240.8 ± 42.04 |
| *L. aboreus* | 28.46 ± 4.29 | 422.2 ± 125.87 | 40.29 ± 9.45 | 175.29 ± 43.9 |
| *M. sativa* | 38.38 ± 6.67 | 690.5 ± 239.87 | 45.61 ± 3.86 | 219.2 ± 27.66 |
| *M. astonii* | 44.45 ± 5.54 | 911.33 ± 253.93 | 54.36 ± 11.45 | 253.5 ± 68.39 |
| *M. complexa* | 32.78 ± 7.97 | 496.6 ± 213.87 | 45.95 ± 7.41 | 223 ± 40.32 |
| *O. virgata* | 42 ± 7.12 | 710.5 ± 256.08 | 50.6 ± 11.13 | 234.57 ± 64.25 |
| *O. leptophyllus* | 48.6 ± 7.7 | 901.67 ± 237.53 | 44.52 ± 4.44 | 206.43 ± 23.61 |
| *P. cookianum* | 42.89 ± 12.07 | 786.25 ± 344.18 | 43.62 ± 6.02 | 200.17 ± 32.51 |
| *P. contorta* | 38.79 ± 6.21 | 611.57 ± 186.29 | 41.14 ± 5.79 | 183.33 ± 27.29 |
| *P. radiata* | 30.69 ± 5.2 | 430.25 ± 132.1 | 41.76 ± 10.03 | 198.75 ± 50.33 |
| *P. cita* | 42.34 ± 7.18 | 784.4 ± 231.39 | 45.21 ± 6.07 | 201.67 ± 29.55 |
| *P. colensoi* | 39.14 ± 10.89 | 604.5 ± 280.77 | 39.16 ± 3.83 | 182.25 ± 16.5 |
| *P. totara* | 37.1 ± 4.72 | 520 ± 118.18 | 45.17 ± 13.02 | 209 ± 64.32 |
| *R. acetosella* | 37.62 ± 10.65 | 647.4 ± 303.53 | 52.17 ± 5.35 | 233.67 ± 24.54 |
| *R. obtusifolius* | 32.22 ± 12 | 576.33 ± 389.04 | 47.8 ± 5.76 | 231.25 ± 20.04 |
| *S. microphylla* | 36.67 ± 13.18 | 601.33 ± 416.61 | 49.52 ± 10.35 | 233.5 ± 49.07 |
| *T. pratense* | 39.44 ± 10.19 | 733.25 ± 318.63 | 53.74 ± 9.08 | 248.67 ± 59.34 |
| *T. repens* | 35.53 ± 6.92 | 514.5 ± 129.4 | 40.38 ± 7.32 | 194.33 ± 47.54 |
| *U. europaeus* | 32.08 ± 9.89 | 478.6 ± 297.96 | 44.55 ± 9.98 | 213 ± 58.32 |

**Table S3. The mean ± SD Faith's diversity and species richness of fungal and bacterial soil communities are summarized by the genus-level identity of their respective plant host.**

| **Plant genus** | **Bacterial communities** | | **Fungal communities** | |
| --- | --- | --- | --- | --- |
|  | **Faith's diversity** | **Species richness** | **Faith's diversity** | **Species richness** |
| *Acacia* | 31.08 ± 7.2 | 502 ± 230.54 | 40.08 ± 4.81 | 191.17 ± 24.41 |
| *Acaena* | 31.98 ± 8.56 | 460 ± 231.75 | 43.62 ± 8.93 | 206.1 ± 43.66 |
| *Achillea* | 37.43 ± 5.04 | 631 ± 186.16 | 44.03 ± 6.67 | 212.83 ± 34.52 |
| *Agrostis* | 34.34 ± 7.11 | 475.33 ± 133.87 | 52.88 ± 7.99 | 237 ± 40.27 |
| *Alnus* | 37.59 ± 4.99 | 683.4 ± 178.42 | 43.63 ± 6.93 | 212.17 ± 32.62 |
| *Anemanthele* | 33.61 ± 12.89 | 543 ± 319.74 | 49.69 ± 4.13 | 244.6 ± 21.33 |
| *Brachyglottis* | 42.02 ± 5.35 | 730.67 ± 263.03 | 42.71 ± 6.39 | 199 ± 27.59 |
| *Carex* | 33.51 ± 7.92 | 553.6 ± 245.82 | 45.95 ± 4.56 | 212.17 ± 31.68 |
| *Chionochloa* | 39.2 ± 4.71 | 521 ± 115.97 | 41.29 ± 5.88 | 178.33 ± 24.11 |
| *Cirsium* | 32.5 ± 6.16 | 479 ± 121.53 | 47.92 ± 5.89 | 223.71 ± 29.49 |
| *Coprosma* | 39.24 ± 5.13 | 640.67 ± 176.79 | 39.74 ± 10.42 | 188 ± 49.2 |
| *Dactylis* | 30.89 ± 10.57 | 448 ± 280.33 | 44.28 ± 10.75 | 216.75 ± 55.33 |
| *Echium* | 35.6 ± 11.55 | 671.2 ± 411.52 | 51.75 ± 8.48 | 244.5 ± 48.94 |
| *Festuca* | 42.77 ± 9.37 | 767.8 ± 335.18 | 43.5 ± 6.39 | 203 ± 27.68 |
| *Hebe* | 37.61 ± 12.43 | 608.8 ± 397.97 | 51.05 ± 5.69 | 239.2 ± 26.32 |
| *Holcus* | 38.26 ± 12.8 | 676 ± 347.42 | 54.2 ± 8.86 | 260.83 ± 45.16 |
| *Hypericum* | 38.16 ± 7.81 | 699.33 ± 247.23 | 45.37 ± 9.82 | 220.17 ± 52.57 |
| *Lolium* | 47.32 ± 8.02 | 963 ± 268.71 | 49.7 ± 6.21 | 240.8 ± 42.04 |
| *Lupinus* | 28.46 ± 4.29 | 422.2 ± 125.87 | 40.29 ± 9.45 | 175.29 ± 43.9 |
| *Medicago* | 38.38 ± 6.67 | 690.5 ± 239.87 | 45.61 ± 3.86 | 219.2 ± 27.66 |
| *Muehlenbeckia* | 39.15 ± 8.82 | 722.82 ± 312.16 | 50.15 ± 10.19 | 238.25 ± 55.85 |
| *Olearia* | 42 ± 7.12 | 710.5 ± 256.08 | 50.6 ± 11.13 | 234.57 ± 64.25 |
| *Ozothamnus* | 48.6 ± 7.7 | 901.67 ± 237.53 | 44.52 ± 4.44 | 206.43 ± 23.61 |
| *Phormium* | 42.89 ± 12.07 | 786.25 ± 344.18 | 43.62 ± 6.02 | 200.17 ± 32.51 |
| *Pinus* | 35.84 ± 6.92 | 545.64 ± 185.54 | 41.33 ± 6.9 | 188.08 ± 34.42 |
| *Poa* | 40.92 ± 8.55 | 704.44 ± 255.58 | 42.79 ± 5.93 | 193.9 ± 26.01 |
| *Podocarpus* | 37.1 ± 4.72 | 520 ± 118.18 | 45.17 ± 13.02 | 209 ± 64.32 |
| *Rumex* | 35.59 ± 10.67 | 620.75 ± 311.84 | 49.67 ± 5.62 | 232.29 ± 20.08 |
| *Sophora* | 36.67 ± 13.18 | 601.33 ± 416.61 | 49.52 ± 10.35 | 233.5 ± 49.07 |
| *Trifolium* | 38.14 ± 8.71 | 660.33 ± 277.53 | 49.28 ± 10.47 | 230.56 ± 59.19 |
| *Ulex* | 32.08 ± 9.89 | 478.6 ± 297.96 | 44.55 ± 9.98 | 213 ± 58.32 |

**Table S4. The mean ± SD Faith's diversity and species richness of fungal and bacterial soil communities are summarized by the family-level identity of their respective plant host.**

| **Plant family** | **Bacterial communities** | | **Fungal communities** | |
| --- | --- | --- | --- | --- |
|  | **Faith's diversity** | **Species richness** | **Faith's diversity** | **Species richness** |
| Asphodelaceae | 42.89 ± 12.07 | 786.25 ± 344.18 | 43.62 ± 6.02 | 200.17 ± 32.51 |
| Asteraceae | 40.91 ± 8 | 700.25 ± 248.84 | 46.01 ± 7.44 | 215.38 ± 38.69 |
| Betulaceae | 37.59 ± 4.99 | 683.4 ± 178.42 | 43.63 ± 6.93 | 212.17 ± 32.62 |
| Boraginaceae | 35.6 ± 11.55 | 671.2 ± 411.52 | 51.75 ± 8.48 | 244.5 ± 48.94 |
| Cyperaceae | 33.51 ± 7.92 | 553.6 ± 245.82 | 45.95 ± 4.56 | 212.17 ± 31.68 |
| Fabaceae | 34.11 ± 9.07 | 557.75 ± 278.05 | 45.1 ± 9.17 | 210.82 ± 49.15 |
| Hypericaceae | 38.16 ± 7.81 | 699.33 ± 247.23 | 45.37 ± 9.82 | 220.17 ± 52.57 |
| Pinaceae | 35.84 ± 6.92 | 545.64 ± 185.54 | 41.33 ± 6.9 | 188.08 ± 34.42 |
| Plantaginaceae | 37.61 ± 12.43 | 608.8 ± 397.97 | 51.05 ± 5.69 | 239.2 ± 26.32 |
| Poaceae | 39.4 ± 10.03 | 676.61 ± 298.26 | 47.14 ± 7.95 | 221.33 ± 42.26 |
| Podocarpaceae | 37.1 ± 4.72 | 520 ± 118.18 | 45.17 ± 13.02 | 209 ± 64.32 |
| Polygonaceae | 37.65 ± 9.53 | 679.84 ± 307.63 | 49.98 ± 8.6 | 236.05 ± 45.27 |
| Rosaceae | 31.98 ± 8.56 | 460 ± 231.75 | 43.62 ± 8.93 | 206.1 ± 43.66 |
| Rubiaceae | 39.24 ± 5.13 | 640.67 ± 176.79 | 39.74 ± 10.42 | 188 ± 49.2 |

**Table S5. The mean ± SD Faith's diversity and species richness of fungal and bacterial soil communities are summarized by the order-level identity of their respective plant host.**

| **Plant order** | **Bacterial communities** | | **Fungal communities** | |
| --- | --- | --- | --- | --- |
|  | **Faith's diversity** | **Species richness** | **Faith's diversity** | **Species richness** |
| Asparagales | 42.89 ± 12.07 | 786.25 ± 344.18 | 43.62 ± 6.02 | 200.17 ± 32.51 |
| Asterales | 40.91 ± 8 | 700.25 ± 248.84 | 46.01 ± 7.44 | 215.38 ± 38.69 |
| Boraginales | 35.6 ± 11.55 | 671.2 ± 411.52 | 51.75 ± 8.48 | 244.5 ± 48.94 |
| Caryophyllales | 37.65 ± 9.53 | 679.84 ± 307.63 | 49.98 ± 8.6 | 236.05 ± 45.27 |
| Fabales | 34.11 ± 9.07 | 557.75 ± 278.05 | 45.1 ± 9.17 | 210.82 ± 49.15 |
| Fagales | 37.59 ± 4.99 | 683.4 ± 178.42 | 43.63 ± 6.93 | 212.17 ± 32.62 |
| Gentianales | 39.24 ± 5.13 | 640.67 ± 176.79 | 39.74 ± 10.42 | 188 ± 49.2 |
| Lamiales | 37.61 ± 12.43 | 608.8 ± 397.97 | 51.05 ± 5.69 | 239.2 ± 26.32 |
| Malpighiales | 38.16 ± 7.81 | 699.33 ± 247.23 | 45.37 ± 9.82 | 220.17 ± 52.57 |
| Pinales | 36.24 ± 6.19 | 537.63 ± 163.79 | 42.4 ± 8.75 | 193.89 ± 43.62 |
| Poales | 38.69 ± 9.9 | 661.61 ± 292.48 | 46.99 ± 7.58 | 220.19 ± 40.92 |
| Rosales | 31.98 ± 8.56 | 460 ± 231.75 | 43.62 ± 8.93 | 206.1 ± 43.66 |

**Table S6. The mean ± SD Faith's diversity and species richness of fungal and bacterial soil communities are summarized by the class-level identity of their respective plant host.**

| **Plant class** | **Bacterial communities** | | **Fungal communities** | |
| --- | --- | --- | --- | --- |
|  | **Faith's diversity** | **Species richness** | **Faith's diversity** | **Species richness** |
| Liliopsida | 42.89 ± 12.07 | 786.25 ± 344.18 | 43.62 ± 6.02 | 200.17 ± 32.51 |
| Magnoliopsida | 37.47 ± 9.22 | 637.79 ± 280.81 | 46.43 ± 8.36 | 218.34 ± 43.71 |
| Pinopsida | 36.24 ± 6.19 | 537.63 ± 163.79 | 42.4 ± 8.75 | 193.89 ± 43.62 |

**Table S7. The mean ± SD Faith's diversity and species richness of fungal and bacterial soil communities are summarized by the life span of their respective plant host.**

| **Life span** | **Bacterial communities** | | **Fungal communities** | |
| --- | --- | --- | --- | --- |
|  | **Faith's diversity** | **Species richness** | **Faith's diversity** | **Species richness** |
| Annual | 34.05 ± 8.88 | 575.1 ± 303.46 | 49.69 ± 7.16 | 233.31 ± 39.36 |
| Long-lived | 36.56 ± 5.83 | 572.33 ± 174.74 | 42.71 ± 8.21 | 198.46 ± 41.27 |
| Perennial | 37.85 ± 9.42 | 644.63 ± 284.47 | 46.17 ± 8.39 | 216.71 ± 44.04 |

**Table S8. The mean ± SD Faith's diversity and species richness of fungal and bacterial soil communities are summarized by the N_2_ fixation status of their respective plant host.**

| **N_2_ fixation** | **Bacterial communities** | | **Fungal communities** | |
| --- | --- | --- | --- | --- |
|  | **Faith's diversity** | **Species richness** | **Faith's diversity** | **Species richness** |
| No | 38.25 ± 9.02 | 647.32 ± 275.53 | 46.29 ± 8.26 | 216.9 ± 43.06 |
| Yes | 34.58 ± 8.67 | 574.73 ± 268.34 | 44.9 ± 8.84 | 211 ± 46.94 |

**Table S9. The mean ± SD Faith's diversity and species richness of fungal and bacterial soil communities are summarized by the primary mycorrhizal association of their respective plant host.**

| **Mycorrhizal association** | **Bacterial communities** | | **Fungal communities** | |
| --- | --- | --- | --- | --- |
|  | **Faith's diversity** | **Species richness** | **Faith's diversity** | **Species richness** |
| AMF | 38.18 ± 9.24 | 640.25 ± 277.98 | 46.02 ± 8.32 | 216.44 ± 43.11 |
| EMF | 36.39 ± 6.27 | 588.69 ± 189.18 | 42.06 ± 6.8 | 195.68 ± 34.89 |
| No | 35.39 ± 9.29 | 622.12 ± 300.73 | 47.84 ± 8.9 | 222.42 ± 48.79 |

**Table S10. The mean ± SD Faith's diversity and species richness of fungal and bacterial soil communities are summarized by their respective plant host’s provenance to Aotearoa New Zealand.**

| **Provenance** | **Bacterial communities** | | **Fungal communities** | |
| --- | --- | --- | --- | --- |
|  | **Faith's diversity** | **Species richness** | **Faith's diversity** | **Species richness** |
| Exotic | 35.85 ± 8.6 | 605.66 ± 262.32 | 46.16 ± 8.46 | 217.34 ± 44.61 |
| Native | 39.07 ± 9.24 | 657.87 ± 285.74 | 45.81 ± 8.36 | 213.82 ± 43.27 |

**Table S11. The mean ± SD Faith's diversity and species richness of fungal and bacterial soil communities are summarized by the functional group of their respective plant host.**

| **Functional group** | **Bacterial communities** | | **Fungal communities** | |
| --- | --- | --- | --- | --- |
|  | **Faith's diversity** | **Species richness** | **Faith's diversity** | **Species richness** |
| Forb | 37.92 ± 9.16 | 654.23 ± 276.84 | 46.19 ± 7.56 | 217.47 ± 39.17 |
| Grass | 38.69 ± 9.9 | 661.61 ± 292.48 | 46.99 ± 7.58 | 220.19 ± 40.92 |
| Shrub | 37.02 ± 9.44 | 618.5 ± 299.02 | 47.06 ± 10.28 | 219.79 ± 54.48 |
| Tree | 35.34 ± 6.44 | 556.7 ± 186 | 42.18 ± 7.65 | 197 ± 38.24 |


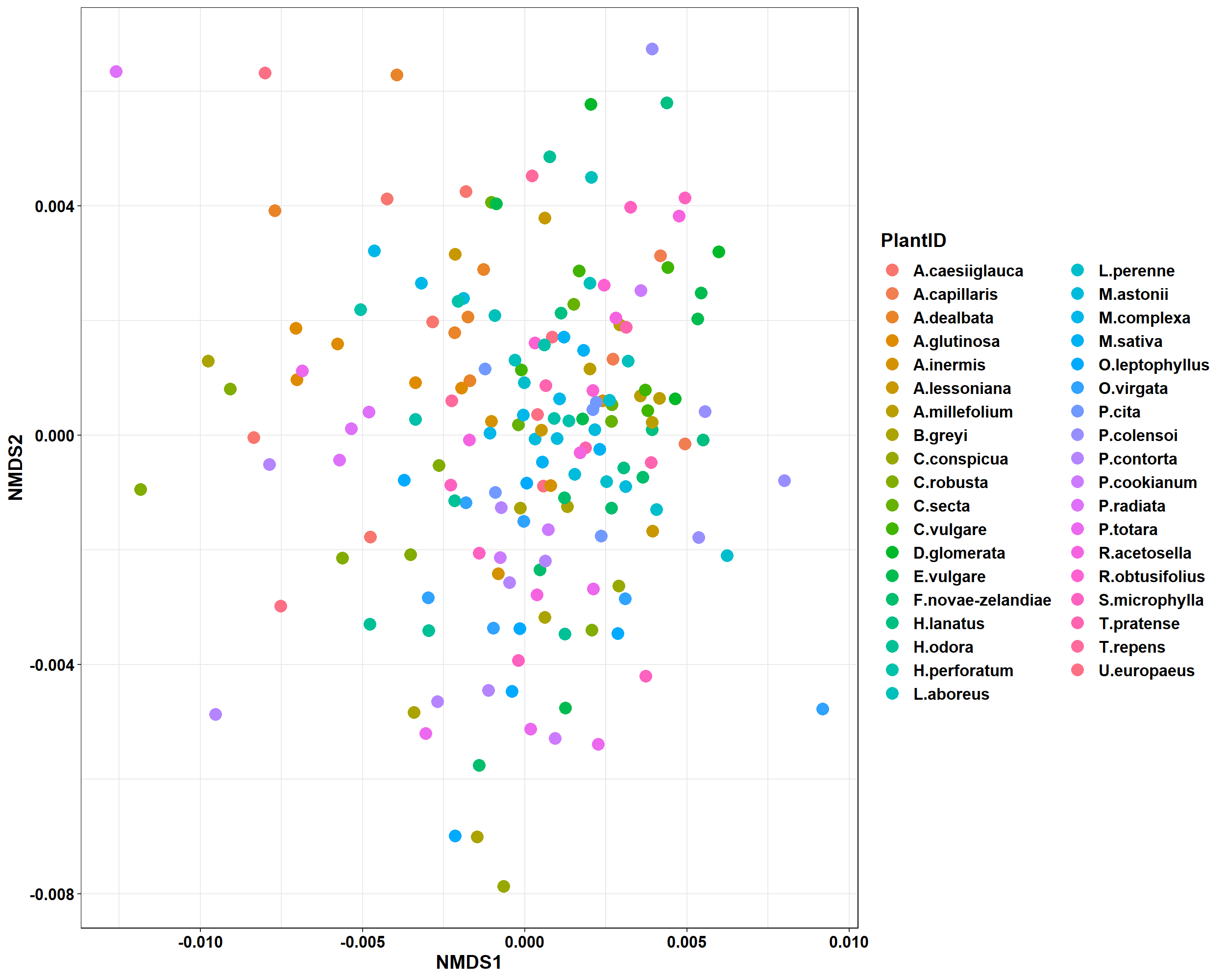


Figure S3. NMDS ordination plots displaying the differences in bacterial community composition across the different plant species. Note: community composition was ordinated by plant species as this was the factor that explained the largest proportion of community variance (as determined using PERMANOVA).


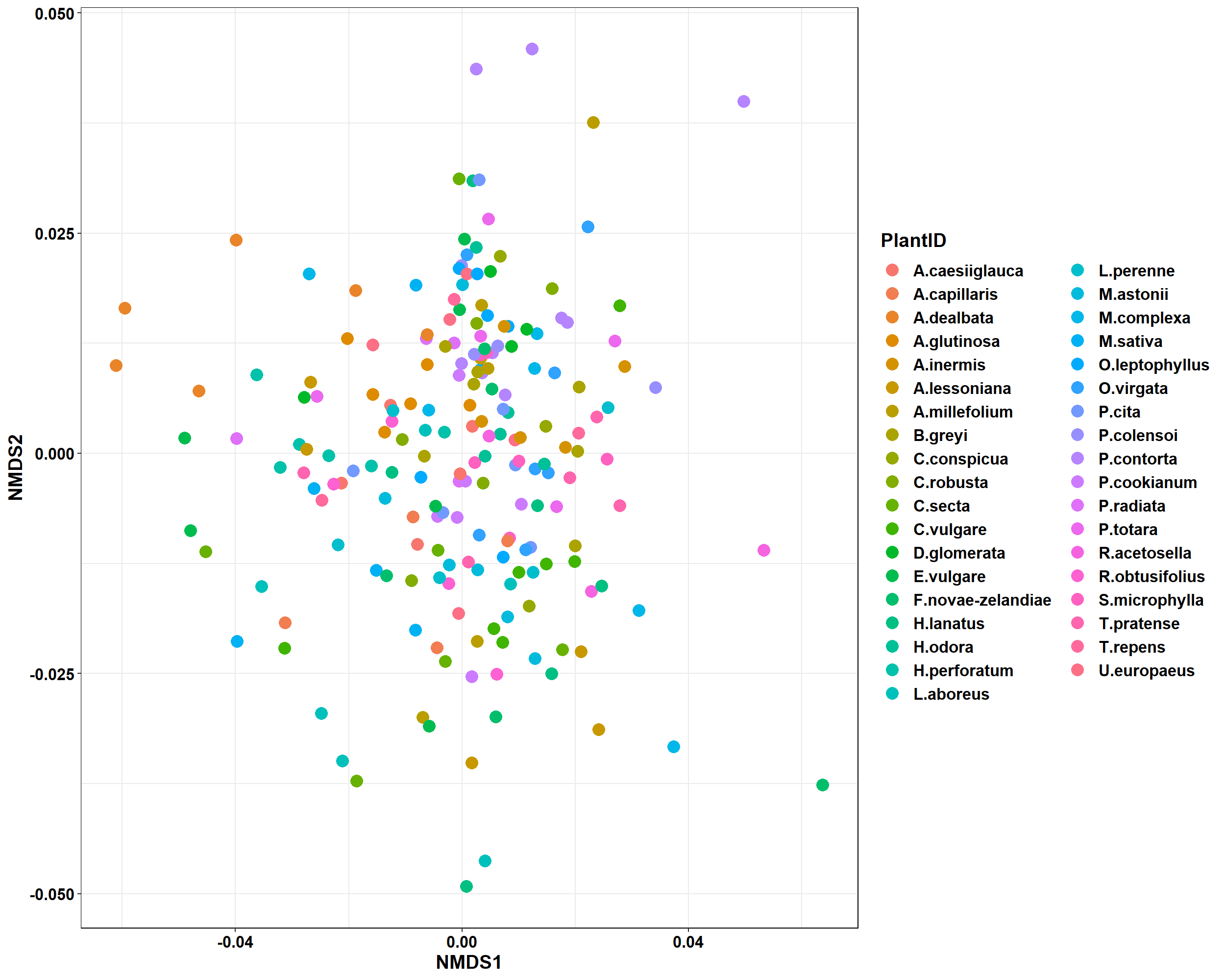


Figure S4. NMDS ordination plots displaying the differences in fungal community composition across the different plant species. Note: community composition was ordinated by plant species as this was the factor that explained the largest proportion of community variance (as determined using PERMANOVA).


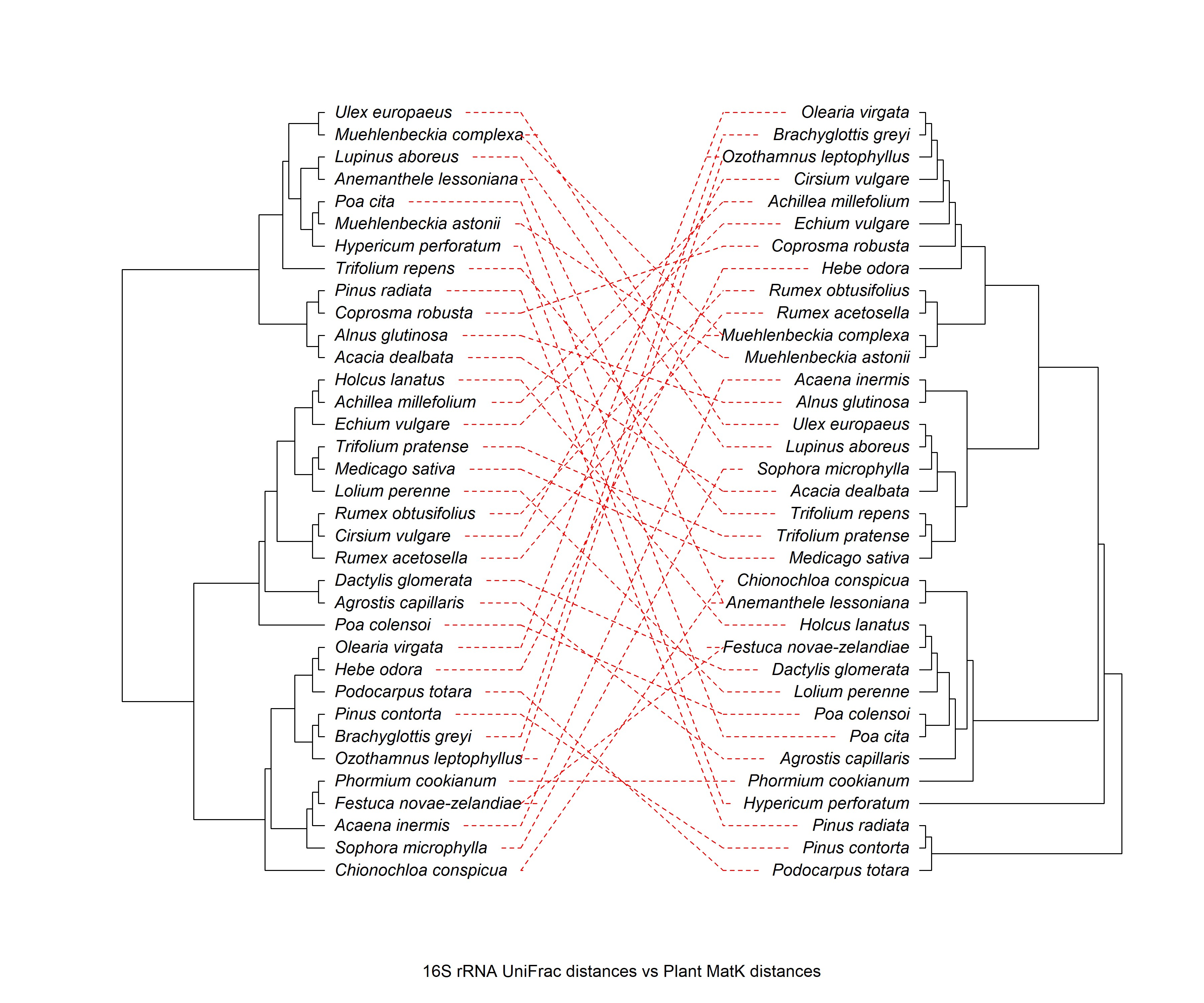


Figure S5. The hierarchical clustering patterns of the plant species based on the weighted UniFrac distances of their bacterial ASVs (left) versus their matK gene sequence similarity (right).


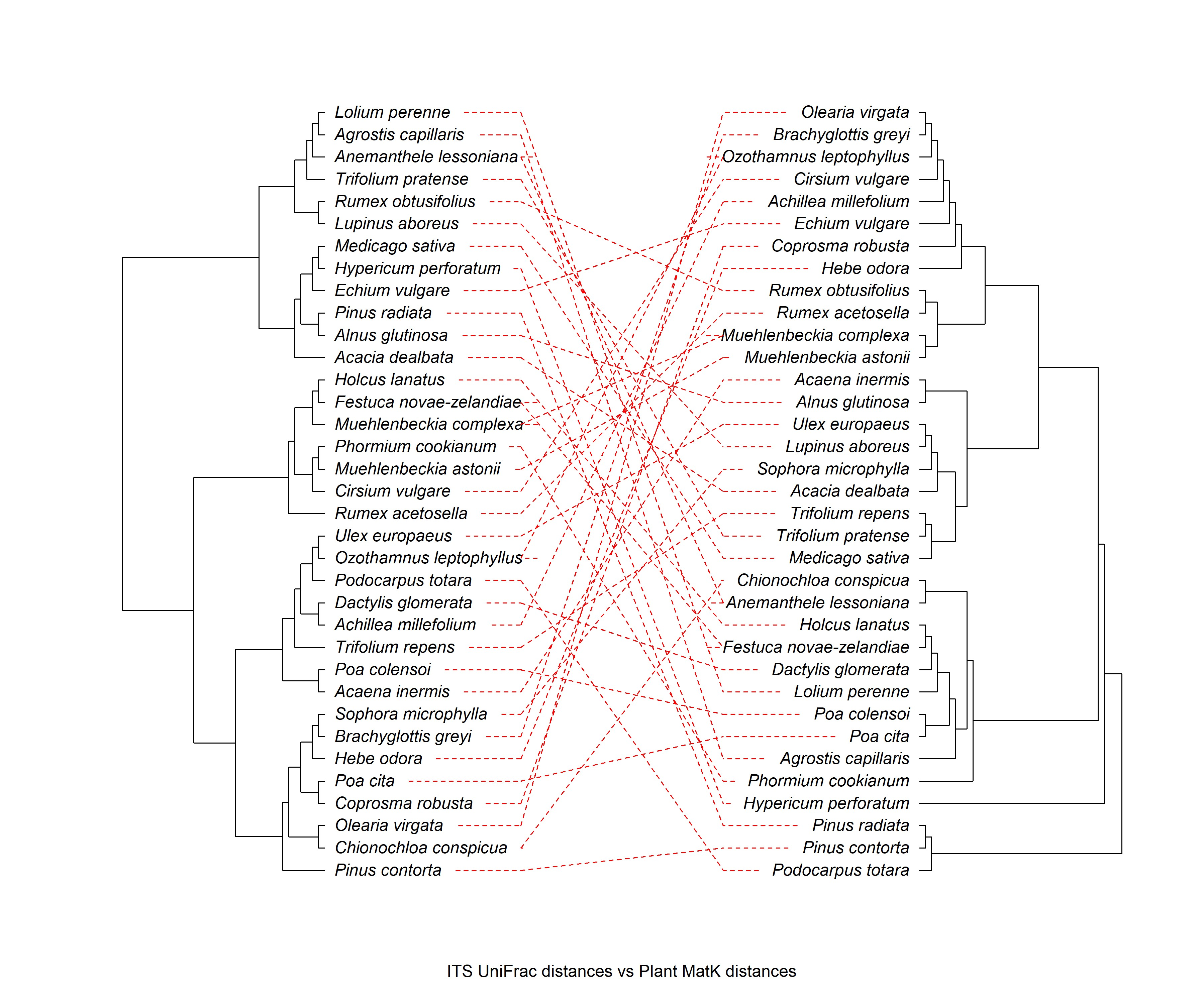


Figure S6. The hierarchical clustering patterns of the different plant species based on the weighted UniFrac distances of their fungal ASVs (left) versus their matK gene sequence similarity (right).

**Table S12. The mean ± SD values for the soil physicochemical properties which were measured for each plant in the experiment. Values have been averaged according to the species-level identity of the plant host. AMN = anaerobically mineralizable N.**

| **Plant species** | **pH** | **Olsen P (mg/L)** | **Sulphate S (mg/kg)** | **Total C (%)** | **Total N (%)** | **C: N ratio** | **Potentially available N (kg/ha)** | **AMN (μg/g)** | **AMN: Total N ratio** | **Vol. weight (g/mL)** |
| --- | --- | --- | --- | --- | --- | --- | --- | --- | --- | --- |
| *A. dealbata* | 5.55 ± 0.10 | 34.83 ± 7.00 | 22.00 ± 7.59 | 2.20 ± 0.23 | 0.20 ± 0.02 | 11.15 ± 0.52 | 61.83 ± 5.08 | 35.67 ± 3.08 | 1.82 ± 0.26 | 1.16 ± 0.04 |
| *A. caesiiglauca* | 5.40 ± 0.21 | 42.80 ± 9.15 | 13.60 ± 5.03 | 2.48 ± 0.19 | 0.20 ± 0.02 | 12.06 ± 0.82 | 73.20 ± 17.67 | 45.20 ± 9.60 | 2.22 ± 0.44 | 1.07 ± 0.03 |
| *A. inermis* | 5.55 ± 0.31 | 28.00 ± 11.97 | 12.75 ± 4.35 | 2.15 ± 0.25 | 0.18 ± 0.02 | 12.30 ± 0.82 | 72.75 ± 11.87 | 42.50 ± 4.80 | 2.43 ± 0.10 | 1.13 ± 0.05 |
| *A. millefolium* | 5.68 ± 0.16 | 16.00 ± 10.46 | 19.60 ± 13.22 | 2.22 ± 0.33 | 0.18 ± 0.02 | 12.44 ± 1.46 | 57.40 ± 29.20 | 33.40 ± 16.99 | 1.84 ± 0.86 | 1.15 ± 0.03 |
| *A. capillaris* | 5.43 ± 0.23 | 32.33 ± 11.15 | 24.30 ± 19.60 | 2.23 ± 0.06 | 0.19 ± 0.02 | 11.60 ± 0.50 | 86.00 ± 5.57 | 51.00 ± 3.00 | 2.67 ± 0.21 | 1.12 ± 0.08 |
| *A. glutinosa* | 5.38 ± 0.11 | 44.20 ± 6.72 | 28.00 ± 7.65 | 2.72 ± 0.75 | 0.22 ± 0.05 | 12.44 ± 0.56 | 65.40 ± 4.72 | 40.20 ± 4.32 | 1.92 ± 0.39 | 1.08 ± 0.07 |
| *A. lessoniana* | 5.63 ± 0.15 | 31.00 ± 1.41 | 16.75 ± 6.13 | 2.33 ± 0.30 | 0.20 ± 0.03 | 11.88 ± 0.43 | 80.25 ± 5.56 | 47.50 ± 4.36 | 2.43 ± 0.26 | 1.13 ± 0.04 |
| *B. greyi* | 5.25 ± 0.19 | 28.50 ± 14.83 | 15.33 ± 6.89 | 2.32 ± 0.22 | 0.20 ± 0.02 | 12.07 ± 1.06 | 63.17 ± 7.31 | 38.50 ± 4.97 | 2.02 ± 0.23 | 1.09 ± 0.06 |
| *C. secta* | 5.68 ± 0.13 | 21.20 ± 6.76 | 14.80 ± 9.28 | 2.26 ± 0.11 | 0.18 ± 0.02 | 12.52 ± 0.74 | 64.60 ± 22.14 | 37.80 ± 13.81 | 2.10 ± 0.88 | 1.14 ± 0.07 |
| *C. conspicua* | 5.65 ± 0.21 | 21.50 ± 6.36 | 8.00 ± 1.41 | 2.35 ± 0.07 | 0.18 ± 0.00 | 12.80 ± 0.14 | 67.50 ± 10.61 | 42.00 ± 4.24 | 2.35 ± 0.21 | 1.06 ± 0.06 |
| *C. vulgare* | 5.35 ± 0.31 | 38.50 ± 13.00 | 15.25 ± 11.59 | 2.33 ± 0.10 | 0.20 ± 0.02 | 11.90 ± 0.79 | 88.50 ± 27.50 | 55.75 ± 18.55 | 2.83 ± 0.90 | 1.07 ± 0.04 |
| *C. robusta* | 5.32 ± 0.26 | 36.50 ± 13.40 | 15.83 ± 7.22 | 2.30 ± 0.17 | 0.19 ± 0.02 | 12.43 ± 1.27 | 61.17 ± 16.46 | 35.17 ± 10.01 | 1.90 ± 0.53 | 1.16 ± 0.08 |
| *D. glomerata* | 5.87 ± 0.15 | 15.67 ± 14.15 | 4.67 ± 4.62 | 2.40 ± 0.35 | 0.20 ± 0.02 | 11.93 ± 0.74 | 93.00 ± 36.40 | 55.70 ± 21.5 | 2.70 ± 0.79 | 1.12 ± 0.10 |
| *E. vulgare* | 5.60 ± 0.19 | 18.80 ± 11.45 | 14.20 ± 7.53 | 2.18 ± 0.33 | 0.17 ± 0.03 | 13.02 ± 0.64 | 59.20 ± 20.87 | 35.00 ± 11.38 | 2.06 ± 0.49 | 1.12 ± 0.05 |
| *F. novae-zelandiae* | 5.72 ± 0.15 | 25.40 ± 10.92 | 6.40 ± 1.14 | 2.14 ± 0.31 | 0.17 ± 0.02 | 12.22 ± 0.91 | 69.80 ± 18.86 | 40.80 ± 12.68 | 2.34 ± 0.50 | 1.14 ± 0.06 |
| *H. odora* | 5.42 ± 0.13 | 31.80 ± 5.17 | 21.00 ± 5.92 | 2.66 ± 0.37 | 0.22 ± 0.02 | 12.16 ± 0.75 | 72.60 ± 10.64 | 47.20 ± 6.14 | 2.18 ± 0.16 | 1.02 ± 0.03 |
| *H. lanatus* | 5.82 ± 0.18 | 20.20 ± 9.58 | 8.60 ± 7.23 | 2.42 ± 0.52 | 0.19 ± 0.03 | 12.40 ± 0.77 | 73.40 ± 15.13 | 41.20 ± 8.47 | 2.16 ± 0.50 | 1.19 ± 0.04 |
| *H. perforatum* | 5.38 ± 0.32 | 31.67 ± 9.85 | 24.00 ± 11.49 | 2.55 ± 0.36 | 0.22 ± 0.03 | 11.82 ± 0.58 | 62.70 ± 25.70 | 37.83 ± 15.79 | 1.78 ± 0.77 | 1.10 ± 0.04 |
| *L. perenne* | 5.64 ± 0.21 | 31.00 ± 6.60 | 11.60 ± 6.54 | 2.30 ± 0.20 | 0.18 ± 0.02 | 12.88 ± 0.36 | 76.40 ± 6.35 | 46.00 ± 4.18 | 2.54 ± 0.15 | 1.11 ± 0.08 |
| *L. aboreus* | 5.48 ± 0.19 | 28.60 ± 8.85 | 8.40 ± 7.40 | 2.26 ± 0.24 | 0.18 ± 0.03 | 12.68 ± 0.84 | 86.00 ± 16.69 | 49.80 ± 11.19 | 2.82 ± 0.55 | 1.15 ± 0.04 |
| *M. sativa* | 5.53 ± 0.36 | 29.50 ± 7.72 | 14.50 ± 4.20 | 2.83 ± 0.60 | 0.23 ± 0.02 | 12.53 ± 1.73 | 80.50 ± 8.74 | 48.75 ± 5.97 | 2.18 ± 0.21 | 1.11 ± 0.07 |
| *M. astonii* | 5.62 ± 0.12 | 27.83 ± 9.35 | 19.17 ± 12.38 | 2.42 ± 0.25 | 0.20 ± 0.02 | 12.27 ± 0.70 | 75.17 ± 22.34 | 44.00 ± 14.66 | 2.20 ± 0.56 | 1.16 ± 0.11 |
| *M. complexa* | 5.42 ± 0.18 | 38.00 ± 5.70 | 16.60 ± 9.10 | 2.30 ± 0.20 | 0.19 ± 0.02 | 11.74 ± 0.44 | 72.20 ± 16.68 | 40.60 ± 9.26 | 2.10 ± 0.46 | 1.19 ± 0.03 |
| *O. virgata* | 5.40 ± 0.20 | 31.17 ± 7.73 | 12.67 ± 4.97 | 2.30 ± 0.28 | 0.19 ± 0.03 | 12.02 ± 0.27 | 64.67 ± 8.12 | 41.50 ± 6.09 | 2.15 ± 0.30 | 1.05 ± 0.05 |
| *O. leptophyllus* | 5.25 ± 0.10 | 21.17 ± 4.96 | 18.50 ± 6.16 | 2.67 ± 0.23 | 0.21 ± 0.01 | 12.85 ± 1.18 | 74.00 ± 6.57 | 46.67 ± 3.61 | 2.23 ± 0.16 | 1.06 ± 0.09 |
| *P. cookianum* | 5.53 ± 0.22 | 28.75 ± 10.81 | 16.00 ± 7.35 | 2.43 ± 0.28 | 0.20 ± 0.02 | 12.25 ± 0.58 | 70.25 ± 18.66 | 41.25 ± 9.98 | 2.15 ± 0.62 | 1.14 ± 0.03 |
| *P. contorta* | 5.26 ± 0.21 | 33.00 ± 7.81 | 18.40 ± 9.63 | 2.32 ± 0.18 | 0.19 ± 0.01 | 11.82 ± 0.90 | 64.40 ± 16.74 | 39.00 ± 8.51 | 1.96 ± 0.35 | 1.09 ± 0.05 |
| *P. radiata* | 5.18 ± 0.10 | 44.50 ± 7.72 | 28.50 ± 7.59 | 2.15 ± 0.17 | 0.19 ± 0.02 | 11.48 ± 0.59 | 70.25 ± 8.34 | 42.50 ± 5.20 | 2.28 ± 0.33 | 1.10 ± 0.03 |
| *P. cita* | 5.80 ± 0.10 | 21.40 ± 11.44 | 8.60 ± 3.85 | 2.26 ± 0.25 | 0.18 ± 0.02 | 12.42 ± 0.30 | 68.80 ± 6.98 | 40.80 ± 4.15 | 2.28 ± 0.45 | 1.13 ± 0.02 |
| *P. colensoi* | 5.80 ± 0.22 | 15.25 ± 1.71 | 6.75 ± 2.06 | 2.25 ± 0.06 | 0.17 ± 0.01 | 13.35 ± 0.76 | 57.25 ± 8.96 | 35.00 ± 5.72 | 2.13 ± 0.38 | 1.10 ± 0.07 |
| *P. totara* | 5.42 ± 0.26 | 26.60 ± 9.86 | 14.80 ± 9.20 | 2.26 ± 0.29 | 0.19 ± 0.02 | 12.12 ± 0.88 | 72.60 ± 20.82 | 43.80 ± 10.80 | 2.42 ± 0.73 | 1.10 ± 0.08 |
| *R. acetosella* | 5.62 ± 0.08 | 28.60 ± 6.58 | 18.80 ± 6.06 | 2.48 ± 0.24 | 0.21 ± 0.02 | 12.14 ± 0.76 | 88.20 ± 17.33 | 50.60 ± 8.11 | 2.50 ± 0.39 | 1.16 ± 0.04 |
| *R. obtusifolius* | 5.50 ± 0.00 | 31.33 ± 6.66 | 15.33 ± 4.04 | 2.40 ± 0.10 | 0.21 ± 0.01 | 11.30 ± 0.17 | 72.33 ± 11.85 | 43.67 ± 9.07 | 2.07 ± 0.38 | 1.11 ± 0.06 |
| *S. microphylla* | 5.53 ± 0.31 | 27.50 ± 9.16 | 20.17 ± 10.26 | 2.52 ± 0.23 | 0.20 ± 0.02 | 12.62 ± 1.03 | 77.67 ± 10.09 | 49.83 ± 5.23 | 2.52 ± 0.17 | 1.04 ± 0.07 |
| *T. pratense* | 5.53 ± 0.24 | 33.25 ± 7.46 | 17.75 ± 9.03 | 2.50 ± 0.14 | 0.21 ± 0.01 | 12.20 ± 0.49 | 75.50 ± 10.85 | 44.00 ± 6.88 | 2.15 ± 0.33 | 1.14 ± 0.04 |
| *T. repens* | 5.30 ± 0.00 | 31.50 ± 4.95 | 43.00 ± 2.83 | 2.50 ± 0.42 | 0.21 ± 0.01 | 12.00 ± 1.27 | 73.50 ± 7.78 | 45.00 ± 1.41 | 2.20 ± 0.14 | 1.09 ± 0.08 |
| *U. europaeus* | 5.36 ± 0.17 | 21.00 ± 7.65 | 14.40 ± 6.31 | 2.62 ± 0.57 | 0.21 ± 0.04 | 12.64 ± 1.05 | 73.00 ± 17.44 | 43.80 ± 11.95 | 2.08 ± 0.16 | 1.12 ± 0.06 |


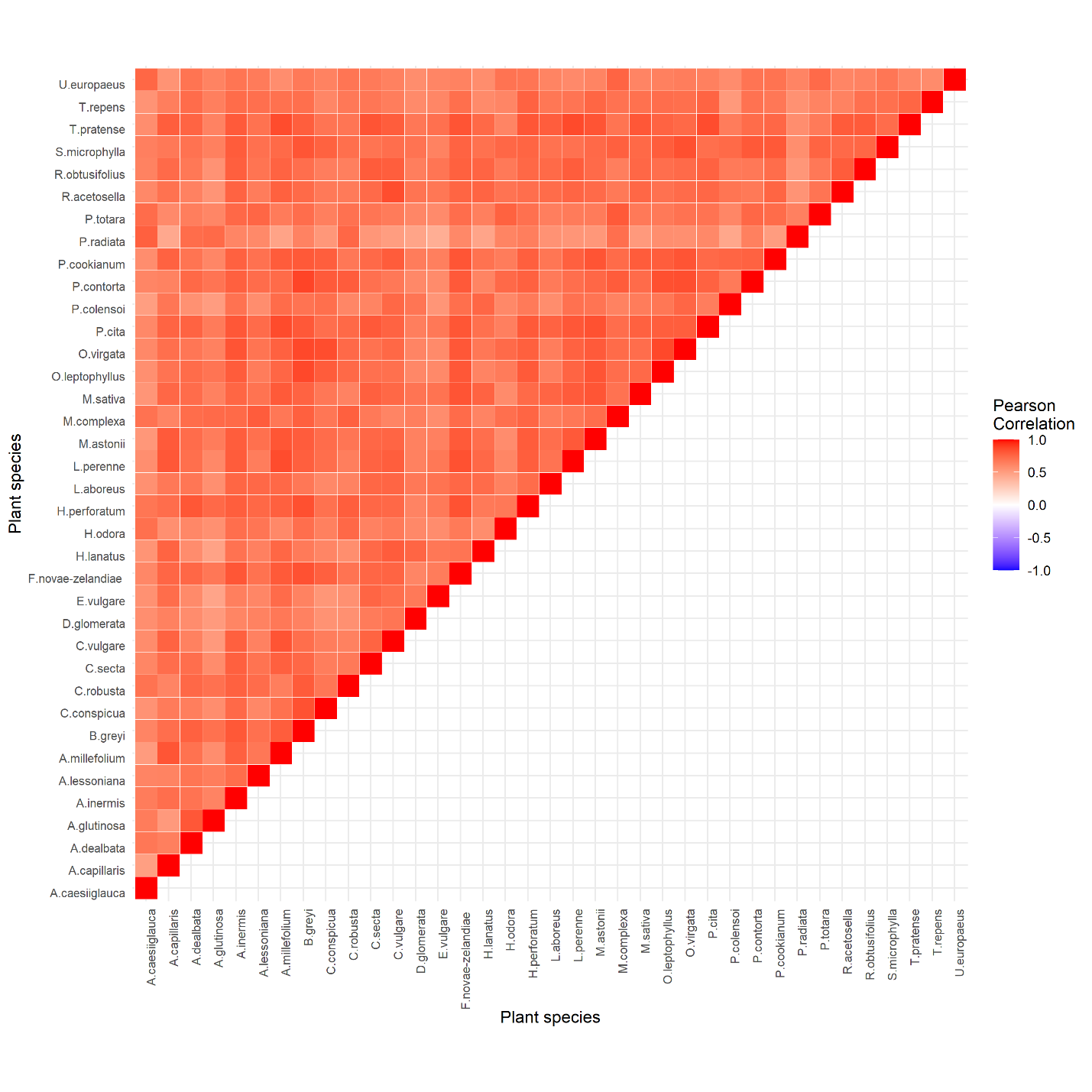


Figure S7. The pairwise Pearson Correlations shared between plant species based on the log change estimates of their bacterial ASVs. All the plant species shared a significant (*p* adjusted < 0.05) positive pairwise correlation based on the abundances of their bacterial ASVs, which indicates that the bacterial microbiomes of each plant species exhibited a low divergence.


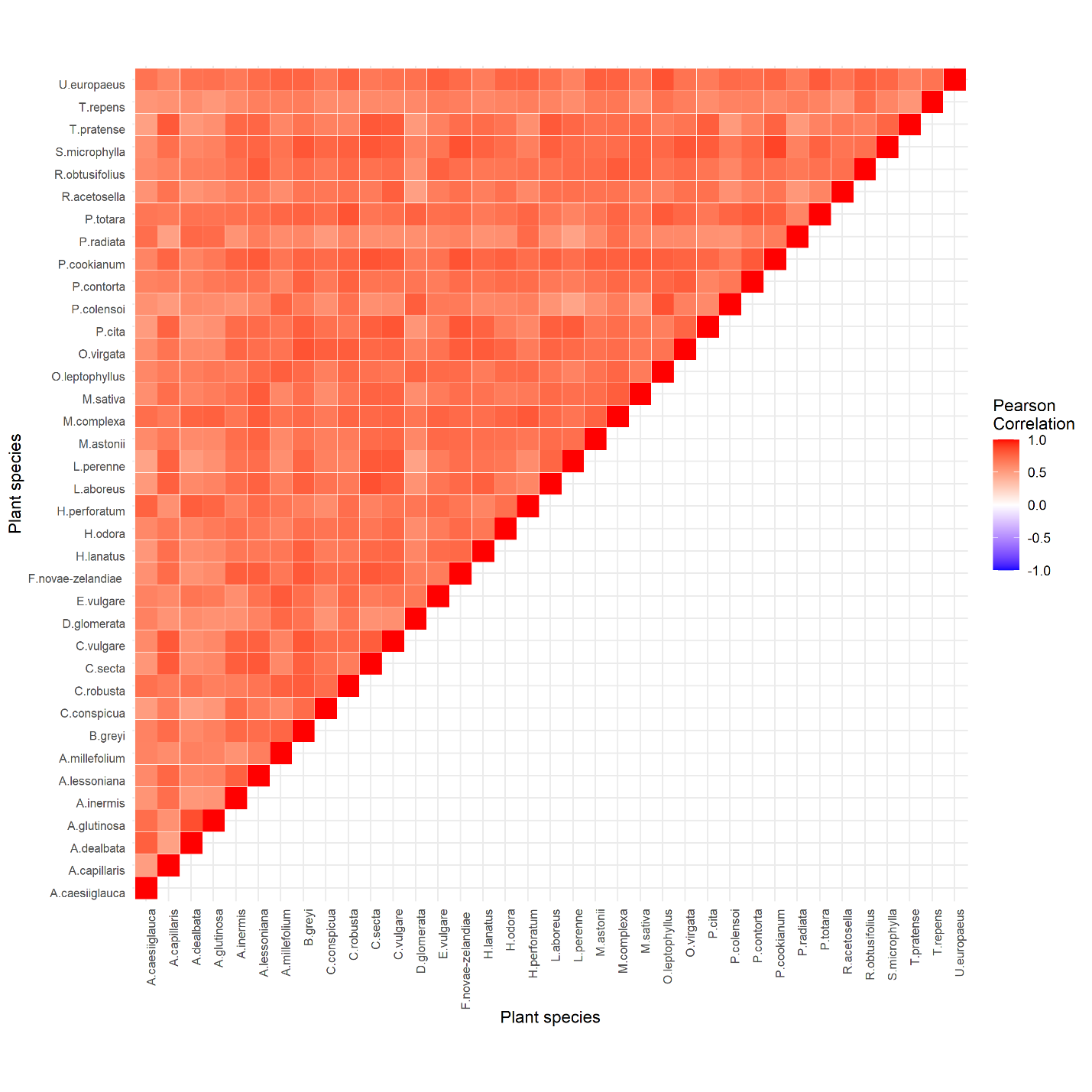


Figure S8. The pairwise Pearson Correlations shared between plant species based on the log change estimates of their fungal ASVs. All the plant species shared a significant (*p* adjusted < 0.05) positive pairwise correlation based on the abundances of their fungal ASVs, which indicates that the fungal microbiomes of each plant species exhibited a low divergence.
